# MicroRNA-200c restoration reveals a cytokine profile to enhance M1 macrophage polarization in breast cancer

**DOI:** 10.1038/s41523-021-00273-1

**Published:** 2021-05-27

**Authors:** Michelle M. Williams, Jessica L. Christenson, Kathleen I. O’Neill, Sabrina A. Hafeez, Claire L. Ihle, Nicole S. Spoelstra, Jill E. Slansky, Jennifer K. Richer

**Affiliations:** 1 Department of Pathology, University of Colorado Anschutz Medical Campus, Aurora, CO USA; 2 Department of Immunology & Microbiology, University of Colorado Anschutz Medical Campus, Aurora, CO USA

**Keywords:** Cancer microenvironment, Tumour immunology

## Abstract

Many immune suppressive mechanisms utilized by triple negative breast cancer (TNBC) are regulated by oncogenic epithelial-to-mesenchymal transition (EMT). How TNBC EMT impacts innate immune cells is not fully understood. To determine how TNBC suppresses antitumor macrophages, we used microRNA-200c (miR-200c), a powerful repressor of EMT, to drive mesenchymal-like mouse mammary carcinoma and human TNBC cells toward a more epithelial state. MiR-200c restoration significantly decreased growth of mouse mammary carcinoma Met-1 cells in culture and in vivo. Cytokine profiling of Met-1 and human BT549 cells revealed that miR-200c upregulated cytokines, such as granulocyte-macrophage colony-stimulating factor (GM-CSF), promoted M1 antitumor macrophage polarization. Cytokines upregulated by miR-200c correlated with an epithelial gene signature and M1 macrophage polarization in BC patients and predicted a more favorable overall survival for TNBC patients. Our findings demonstrate that immunogenic cytokines (e.g., GM-CSF) are suppressed in aggressive TNBC, warranting further investigation of cytokine-based therapies to limit disease recurrence.

## Introduction

Triple negative breast cancer (TNBC) has a higher risk for metastatic spread within 2–3 years after diagnosis when compared to other breast cancer (BC) subtypes^1^. One driver of this early aggressive behavior is oncogenic epithelial-to-mesenchymal transition (EMT). EMT facilitates resistance to chemotherapy, the most common first-line therapy for primary TNBC, and metastasis (reviewed in ref. ^2^). A mesenchymal-to-epithelial transition (MET) often occurs at the metastatic site to allow outgrowth^3^. Therefore, EMT is a plastic process that is also referred to as epithelial-to-mesenchymal plasticity (EMP). Oncogenic EMT is induced by a number of transcription factors, such as zinc finger E-box-binding homeobox 1 (ZEB1) that directly represses transcription of *CDH1*, which encodes the epithelial protein E-cadherin (reviewed in ref. ^4^). Previous studies showed that ZEB1 expression predicts poor BC patient survival^5^ and CDH1 is lost in over 50% of TNBC cases^6^. Circulating tumor cells (CTCs) from patients with TNBC were enriched for mesenchymal markers and the number of mesenchymal-like CTCs further increased with relapse on chemotherapy^7^, demonstrating that the ratio of mesenchymal-to-epithelial CTCs can indicate response to common therapeutic approaches for TNBC.

Oncogenic EMT in breast tumor cells also impacts the tumor microenvironment (TME). For instance, mesenchymal-like mammary tumors had decreased cytotoxic CD8 T-cell activity, possibly due to enhanced tumor cell expression of programmed death-ligand 1 (PD-L1), when compared to epithelial-like mammary tumors^8^. Pertinent to this study, mesenchymal-like mammary tumors also had increased M2 protumor macrophage polarization resulting from enhanced expression of a number of immune-modulatory factors such as colony-stimulating factor-1^9^, a cytokine that supports differentiation and function of protumor macrophages. A pan-EMT signature generated from 11 carcinomas, including BC, revealed that EMT positively correlated with genes encoding immune suppressive factors including PD-L1^10^. Together these studies demonstrate, in both preclinical models and cancer patients, that EMT promotes immune suppression, in part by enhancing checkpoint protein expression. Targeting of PD-L1 and its receptor programmed cell death protein-1 is efficacious in metastatic TNBC^11,12^; however, understanding additional ways that EMT suppresses immune cell infiltration or activation may identify other treatment approaches that enhance response to chemotherapy and/or checkpoint inhibitors.

The microRNA-200 family, known as “guardians of the epithelial phenotype,” directly target and repress many EMT regulators including the mesenchymal transcription factor ZEB1^13^. MicroRNA-200c (miR-200c) negatively correlated with the pan-EMT signature^10^ and is often silenced in TNBC by methylation^14^. Thus, miR-200c restoration is a useful, biologically relevant tool that can be used to potently reverse EMT and thereby reveal factors utilized by TNBC to suppress the TME and facilitate tumor progression. Indeed, restoration of miR-200c in a transgenic claudin-low mouse mammary carcinoma model decreased primary tumor growth and metastasis^15^. Recently, we discovered that restoration of miR-200c to human TNBC cells decreased expression of a number of immune suppressive mechanisms including tryptophan catabolism and checkpoint inhibition via PD-L1^16^, both of which are direct targets of miR-200c^17,18^. In the present study, we developed an in vivo inducible miR-200c model in a cell line derived from a late-stage MMTV-PyMT mouse mammary tumor^19^ in order to further elucidate the role of EMT in suppression of innate immune cells. Previous studies in MMTV-PyMT tumors demonstrated that macrophages are essential for mammary tumor formation^20^ and progression^21^. Further, macrophages are the most abundant immune cell type in the TNBC TME, where their presence predicts poor patient survival^22,23^. Therefore, in the present study, we utilized EMT reversal via miR-200c to identify mechanism(s) utilized by TNBC to suppress macrophage infiltration, polarization, and/or function.

## Results

### Restoration of miR-200c to aggressive mammary carcinoma cells reverses an EMT program and models genes altered by miR-200c in clinical BC specimens

**Fig. 1:**
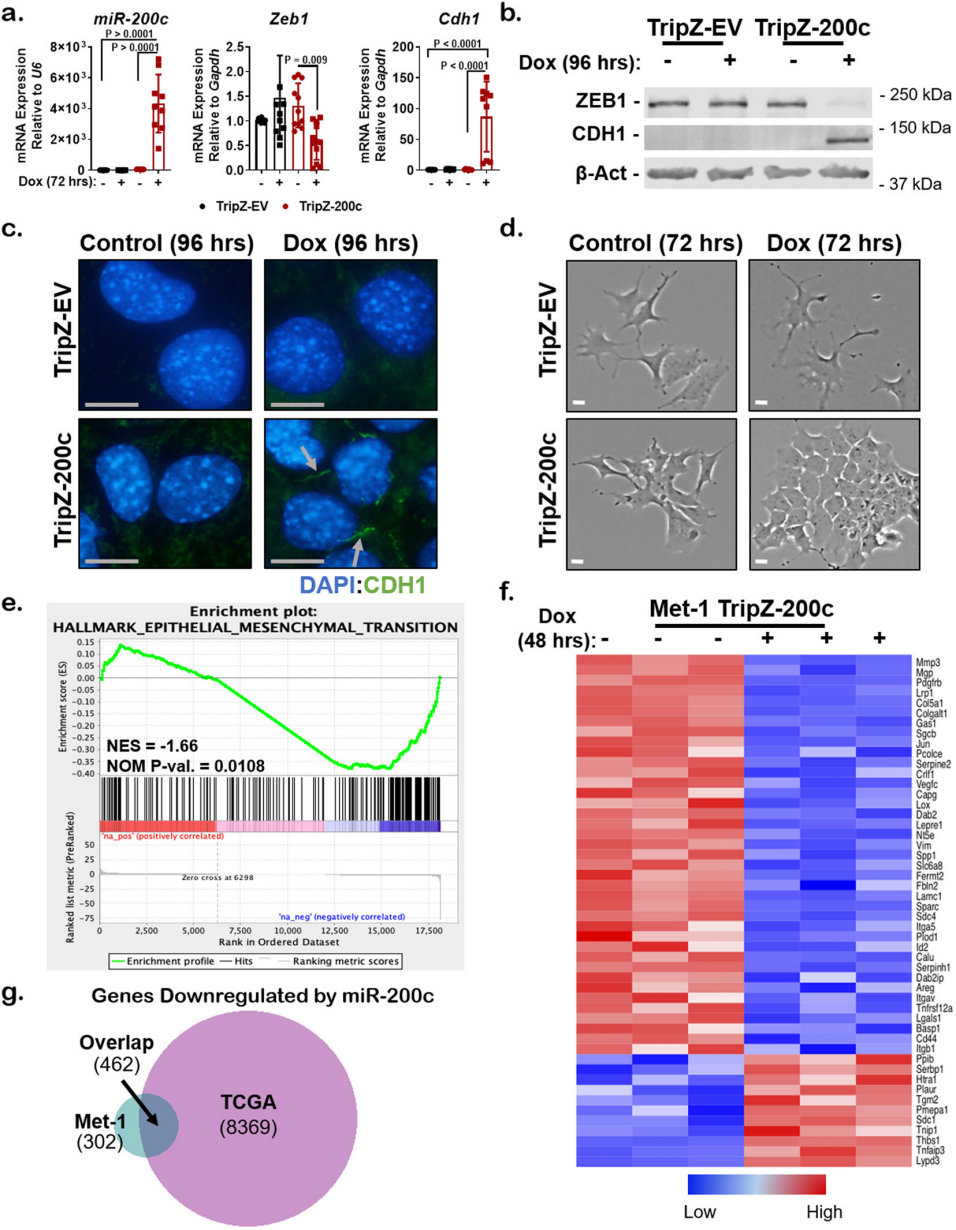
Restoration of miR-200c effectively reverses an epithelial-to-mesenchymal transition (EMT) program in an aggressive mammary carcinoma model. **a** Met-1 TripZ-EV and Met-1 TripZ-200c cells were treated for 72 h with 1.0 µg/mL Dox. Relative expression of *miR-200c* to *U6* and *Zeb1* and *Cdh1* (E-cadherin) to *Gapdh* were determined by qRT-PCR, mean expression of 3–4 experiments conducted in triplicate ± s.d., *N* = 8–12, one-way ANOVA with Tukey’s multiple comparison test. **b**, **c** Met-1 TripZ-EV and Met-1 TripZ-200c cells were treated with 1.0 µg/mL Dox for 96 h. ZEB1, CDH1, and β-Actin were observed by western blot analysis, shown is a representative image, *N* = 3 (**b**). CDH1 localization was observed by immunohistochemistry (ICC; arrows denote membranous CDH1 expression) 100×, scale bar = 10 µM (**c**). **d** Bright field photographs of Met-1 TripZ-EV and Met-1 TripZ-200c cells after treatment with 1.0 µg/mL Dox for 72 h. Shown is a representative image, 4×, scale bar = 30 µM. **e**, **f** mRNA sequencing was conducted on Met-1 TripZ-200c samples from (**a**), *N* = 3 per group. The data were subjected to GSEA analysis (Hallmark), normalized enrichment score (NES), nominal *p* value (NOM *p*-val, **e**). The top 50 genes from the Hallmark-EMT gene set that were altered by miR-200c restoration are presented in a heatmap (**f**). **g** Genes downregulated by miR-200c in Met-1 TripZ-200c cells were compared to genes inversely correlated with miR-200c expression in human BC specimens according to the TCGA (Nature 2012). A corresponding gene list can be seen in Supplementary Data 4.

To further understand whether miR-200c restoration alters immune cell infiltration or activation, a TripZ-empty vector (TripZ-EV) control or TripZ-miR-200c (TripZ-200c) vector was introduced into Met-1 mammary carcinoma cells. Met-1 cells are derived from a late-stage MMTV-PyMT tumor^19^, a transgenic mouse model that closely mimics the morphology and progression of human BC^24^. Although early MMTV-PyMT tumors maintained estrogen receptor (ER) and progesterone receptor expression^24^, expression was lost with progression to late-stage primary tumors and metastases. Met-1 cells have demonstrated negativity for these hormone receptors, but do express androgen receptor, and are a model of ER− disease^25^. Met-1 TripZ-EV or Met-1 TripZ-200c cells that tightly induced the TripZ vector upon doxycycline (Dox) treatment were isolated via cell sorting and used for all experiments (see “Methods”). MiR-200c expression was dramatically increased by Dox treatment in Met-1 TripZ-200c cells, but not in Met-1 TripZ-EV cells (Fig. 1a). The miR-200c target *Zeb1* was significantly decreased by Dox treatment of Met-1 TripZ-200c cells, but not in the Met-1 TripZ-EV controls. The epithelial gene *Cdh1* encoding E-cadherin, which is repressed by ZEB1, was significantly increased in Met-1 TripZ-200c cells after Dox treatment. Western blot confirmed that ZEB1 decreased and CDH1 increased only after Dox treatment of Met-1 TripZ-200c cells (Fig. 1b). Immunocytochemistry (ICC) showed that membranous expression/localization of CDH1 increased in Met-1 TripZ-200c cells after Dox treatment, but not in Met-1 TripZ-EV cells (Fig. 1c). Immunohistochemistry (IHC) demonstrated that nuclear expression of ZEB1 decreased in Met-1 TripZ-200c cells, but not in Met-1 TripZ-EV cells, after Dox treatment (Supplementary Fig. 1a). Further, only the morphology of Met-1 TripZ-200c cells was altered by Dox treatment, which strikingly decreased the spindle-like morphology of cells and instead promoted a less elongated, more epithelial cell shape (Fig. 1d). Changes in additional EMT genes were confirmed by mRNA sequencing of Met-1 TripZ-200c cells treated without or with Dox for 48 h (Supplementary Data 1). Gene Ontology (GO) analysis showed that miR-200c restoration decreased phenotypes like cell migration and EMT and increased phenotypes like cell adhesion (Supplementary Data 2). Similarly, Gene Set Enrichment Analysis (GSEA) showed that the Hallmark-EMT pathway was the pathway most significantly decreased following miR-200c restoration (Supplementary Data 3, Fig. 1e). Analysis of the top 50 Hallmark-EMT genes altered by miR-200c demonstrated consistent downregulation of numerous EMT markers like vimentin (*Vim*) and extracellular matrix and cell adhesion genes like integrin alpha-5 (*Igta5*) (Fig. 1f).

Interestingly, genes downregulated by miR-200c in Met-1 cells showed a >60% overlap with genes that negatively correlated with miR-200c in BC specimens in The Cancer Genome Atlas (TCGA, Fig. 1g). Overlapping genes regulated by miR-200c in both Met-1 cells and BC specimens included EMT genes *ZEB1/*2, *IGTA5*, and *VIM* (Supplementary Data 4), demonstrating that miR-200c regulates similar genes in Met-1 cells and BC specimens. Similar analysis of Met-1 cells compared to a previously published claudin-low mammary tumor model^15^ and a human TNBC cell line^16^ again showed some common genes downregulated by miR-200c (Supplementary Fig. 1b, c and Supplementary Data 5 and 6). These findings demonstrate that Met-1 TripZ-200c cells closely represent genes repressed by miR-200c in BC specimens, thus this model may reveal clinically relevant immune suppressive mechanisms.

### Reversal of EMT via miR-200c alters mammary tumor progression and pathology

**Fig. 2:**
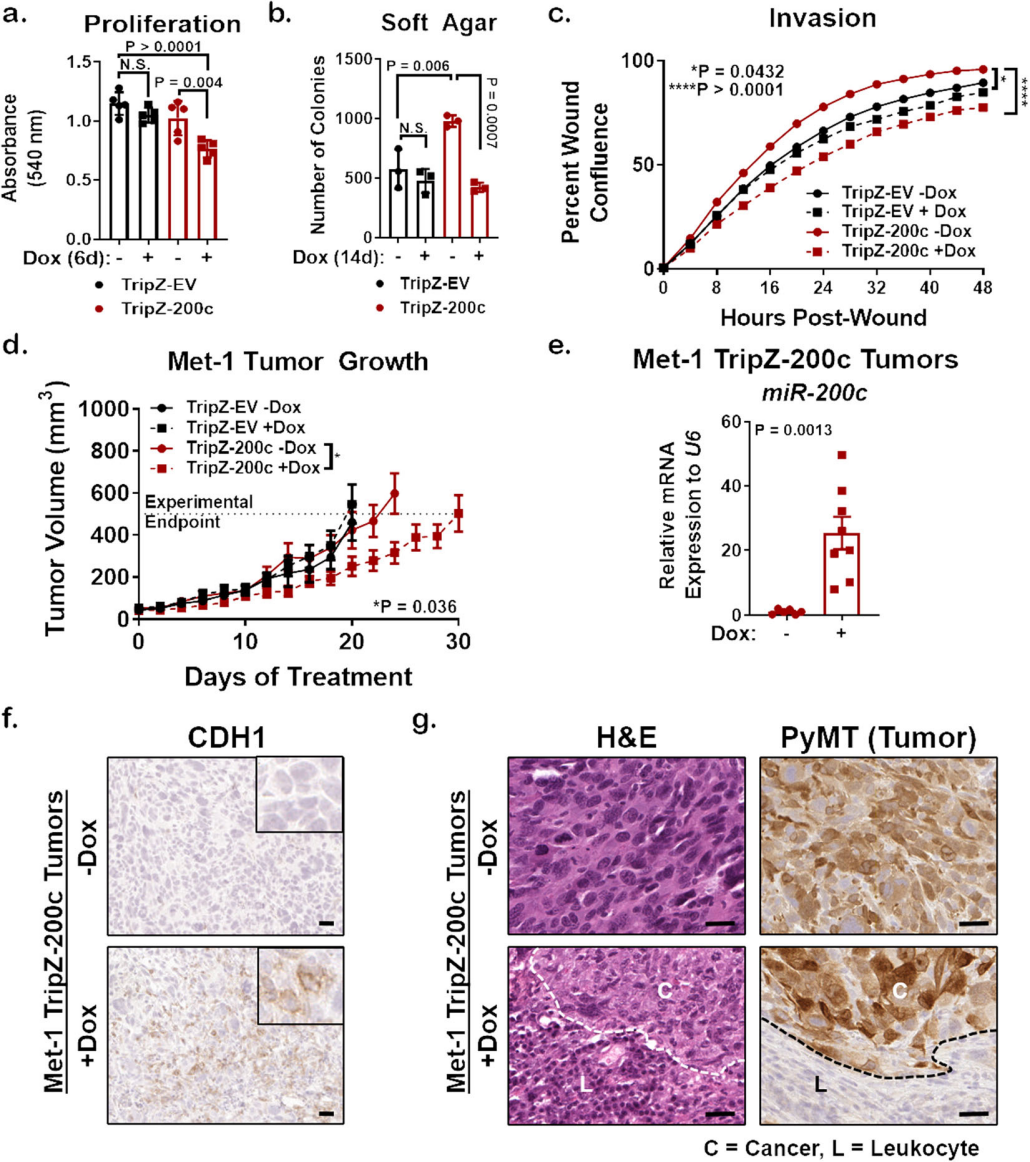
Restoration of miR-200c limits protumor phenotypes in culture, and in vivo delays tumor growth and alters tumor pathology. **a, b** Proliferation (crystal violet staining, **a**) and growth on soft agar (**b**) were determined in Met-1 TripZ-EV and Met-1 TripZ-200c cells after 6 days and 14 days treatment with 1.0 µg/mL Dox, respectively, mean of 3–4 experiments conducted in quintuple or triplicate ± s.d., *N* = 9–15, one-way ANOVA with Tukey’s multiple comparison test. **c** Invasion of Met-1 TripZ-EV and Met-1 TripZ-200c cells treated with 1.0 µg/mL Dox for 48 h was determined using the IncuCyte imaging system, mean percent wound confluence ± s.d., *N* = 10, two-way ANOVA. **d** Met-1 TripZ-EV or Met-1 TripZ-200c cells were orthotopically injected into the mammary fat pads of FVB/NJ syngeneic hosts. When tumor volume reached an average of 50 mm^3^ mice were randomized to treatment with normal chow or Dox chow (3.0 mg/kg/daily). Tumors were harvested when the average tumor volume for each group reached 500 mm^3^. Shown is the mean tumor volume ± s.e.m, *N* = 10–14, two-way ANOVA for all tumors days 0 to 20 was not significant. Shown is the two-way ANOVA conducted on Met-1 TripZ-200c tumors from days 0 to 24. **e** mRNA was extracted from half of each Met-1 TripZ-200c tumor and analyzed by qRT-PCR for *miR-200c*, mean mRNA expression relative to *U6* ± s.e.m, *N* = 6–8, Student’s unpaired two-tailed *t* test. **f**, **g** Half of each Met-1 TripZ-200c tumor was FFPE and analyzed by staining for CDH1 (**f**) and for hematoxylin and eosin (H&E) and PyMT (**g**). Shown is a representative image for each group, 5×—CDH1, 10×—H&E, and PyMT (tumor cell marker), scale bar = 30 µm. Inset for CDH1 is 20×. H&E and PyMT representative images for Met-1 TripZ-200c tumors + miR-200c are stratified for cancer cell dominant (C) and leukocyte dominant (L) regions.

We showed that miR-200c restoration altered numerous EMT genes and proteins, as well as cell morphology. To test whether miR-200c functionally impacted tumor promoting phenotypes, as miR-200c does in human BC cells^26^, Met-1 TripZ-EV and Met-1 TripZ-200c cells were evaluated for proliferation (Fig. 2a), growth on soft agar (Fig. 2b), migration (Supplementary Fig. 2a), and invasion (Fig. 2c). Although Met-1 TripZ-200c cells showed increased tumorigenic properties when compared to Met-1 TripZ-EV cells at baseline (-Dox), Dox treatment in Met-1 TripZ-200c cells alone significantly decreased each one of these phenotypes, suggesting that miR-200c restoration might also affect mammary tumor progression in vivo. We thus injected Met-1 TripZ-EV and Met-1 TripZ-200c cells orthotopically into syngeneic FVB/NJ mice and initiated treatment with Dox, in the diet/chow, when the average tumor volume reached 50 mm^3^. Growth of Met-1 TripZ-EV tumors did not change with Dox treatment (Fig. 2d), demonstrating that Dox alone did not affect tumor progression. Tumor growth was also similar between control Met-1 TripZ-EV and control Met-1 TripZ-200c tumors, suggesting that both cell lines had similar tumorigenic capacities in vivo. However, volume of Met-1 TripZ-200c tumors significantly decreased after miR-200c restoration/Dox treatment when compared to control Met-1 TripZ-200c tumors from mice administered control chow. MiR-200c restoration extended the number of days it took for Met-1 TripZ-200c tumors to reach endpoint (average tumor volume of >500 mm^3^). We confirmed expression of miR-200c in induced Met-1 TripZ-200c tumors via quantitative real-time PCR (qRT-PCR) (Fig. 2e). IHC of formalin fixed paraffin embedded (FFPE) tumors showed increased expression of CDH1 on tumor cell membranes after miR-200c restoration (Fig. 2f). We additionally observed pathology of Met-1 TripZ-200c tumors via hematoxylin and eosin (H&E) staining and were surprised to see infiltration of small round cells that resembled immune cells in tumors from mice treated with Dox/miR-200c restoration (Fig. 2g). Additional analysis of PyMT, a tumor-specific marker in this model, demonstrated that these small round cells were not tumor cells and were, therefore, host-derived. H&E and PyMT staining in Met-1 TripZ-EV tumors confirmed that this phenotype was not due to Dox treatment alone (Supplementary Fig. 2b). Finally, CD8 IHC demonstrated that some of these infiltrating immune cells were cytotoxic T cells that significantly increased in Met-1 TripZ-200c tumors after miR-200c restoration/Dox treatment (Supplementary Fig. 2c).

### MiR-200c restoration enhances secretion of cytokines that enhance M1 antitumor macrophage polarization

**Fig. 3:**
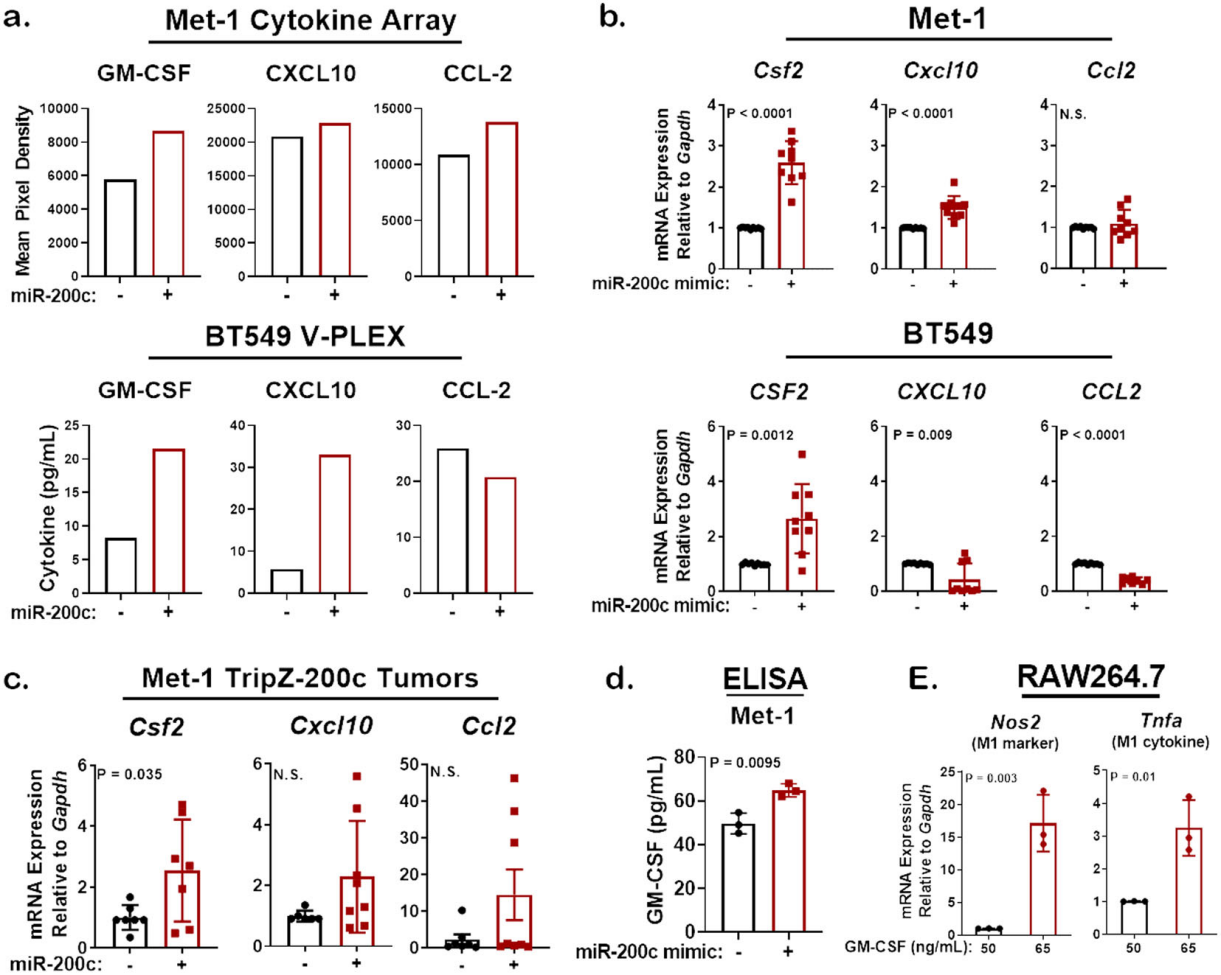
MiR-200c restoration alters the profile of cytokines secreted by aggressive mammary and breast carcinoma cells to favor those that enhance antitumor macrophage polarization. **a** A cytokine array was conducted on Met-1 TripZ-200c cell conditioned medium (CM; mouse) and a V-PLEX cytokine assay was conducted on BT549 TripZ-200c cell CM (human). Depicted are cytokines of interest that were altered in both models after miR-200c restoration and are known to promote monocyte infiltration, activation, or differentiation. **b** Met-1 (top) and BT549 (bottom) cells were harvested after 96 and 48 h, respectively, transient miR-200c restoration. qRT-PCR was conducted for *CSF2* (GM-CSF), *CXCL10*, and *CCL2*, mean mRNA expression relative to *GAPDH* for 3 experiments conducted in triplicate ± s.d., *N* = 8–9, Student’s unpaired two-tailed *t* test. **c** qRT-PCR was conducted on Met-1 TripZ-200c tumors from Fig. 2d that were treated ± Dox for 24 to 30 days. Shown is the mean mRNA expression relative to *Gapdh* ± s.e.m, *N* = 6–8, Students unpaired two-tailed *t* test. **d** GM-CSF expression was observed in the CM of Met-1 cells after miR-200c restoration for 96 h via transient transfection. Shown is the mean cytokine expression (pg/mL) for three experiments completed in triplicate ± s.d., *N* = 9, Students unpaired two-tailed *t* test. **e** RAW264.7 macrophages were treated with 50 ng/mL or 65 ng/mL GM-CSF for 48 h. Cells were harvested for mRNA extraction via TRIzol and macrophage polarization was determined via qRT-PCR for an M1 gene *Nos2* (nitric oxide synthase/iNOS) and a cytokine secreted by M1 macrophages *Tnfa* (tumor necrosis factor-α, TNFα). Representative mean mRNA expression relative to *Gapdh* for one experiment of three ± s.d., *N* = 3, Student’s unpaired two-tailed *t* test.

Cytokines and chemokines are key secreted factors that powerfully alter immune cell infiltration, differentiation, and activation in the TME (reviewed in refs. ^27,28^). To identify tumor cell-secreted cytokines altered by miR-200c restoration, we conducted cytokine profiling on conditioned medium (CM) from mouse Met-1 TripZ-200c and human TNBC BT549 TripZ-200c cells (Supplementary Figs. 3 and 4, respectively). In both the mouse and human models, miR-200c induction via Dox treatment increased expression of granulocyte-macrophage colony-stimulating factor (GM-CSF) and C–X–C motif chemokine 10 (CXCL10) (Fig. 3a). MiR-200c restoration also increased C–C motif chemokine ligand 2 (CCL2) in Met-1 cell CM, but not in human BT549 cell CM. Interestingly, each one of these cytokines alters monocyte infiltration, differentiation, and/or activation, suggesting that restoration of miR-200c to tumor cells may also alter macrophages, a cell type essential for MMTV-PyMT mammary tumor progression^20,21^.

We confirmed miR-200c-dependent upregulation of GM-CSF (*CSF2*), CXCL10, and CCL2 after transient transfection of miR-200c mimic compared to scramble (Scr) control in mouse mammary carcinoma and human TNBC cells (Fig. 3b and Supplementary Fig. 5). qRT-PCR and western blot analysis for CDH1 and ZEB1 confirmed successful miR-200c restoration (Supplementary Fig. 5a–c). MiR-200c restoration enhanced *CSF2* in both Met-1 and BT549 cell lines when compared to Scr control (Fig. 3b). *CXCL10* increased only in Met-1 cells, while *CCL2* decreased in both cell lines after miR-200c restoration. Due to heterogeneity in miR-200c-dependent regulation of these cytokines, we tested two additional cell lines. Transient miR-200c restoration significantly increased *CSF2* in mouse mammary carcinoma 66Cl-4 cells and in human TNBC SUM159PT cells (Supplementary Fig. 5d, e). However, *CXCL10* increased only in SUM159PT cells after miR-200c restoration. Finally, we tested expression of these cytokines in the Met-1 TripZ-200c tumors where miR-200c restoration decreased tumor volume (from Fig. 2). Similar to Met-1 cells in culture, expression of *Csf2* significantly increased in Met-1 TripZ-200c tumors (Fig. 3c). The level of *Cxcl10* and *Ccl2* upregulation was heterogeneous and did not reach significance in Met-1 TripZ-200c tumors.

Since Dox-inducible and transient transfection of miR-200c significantly increased *CSF2* in all models, we measured GM-CSF expression in the CM of Met-1 cells by ELISA. CM from miR-200c transfected cells had significantly increased GM-CSF when compared to CM from Scr control transfected cells (Fig. 3d). GM-CSF treatment is known to polarize macrophages to an antitumor M1 classically activated state^29^. We thus treated RAW264.7 cells, a mouse macrophage cell line originally isolated from the peritoneum of an *Abelson leukemia* virus infected BALB/c mouse^30^, with the same concentration of GM-CSF measured in the CM of Met-1 Scr or Met-1 miR-200c-expressing cells. We chose RAW264.7 cells for this and future studies because they are an established model of macrophage polarization, which can be measured by elevated expression of established genes characteristic of M1 antitumor or M2 protumor phenotypes^31,32^. As expected, an increased dosage of GM-CSF for 48 h significantly induced expression of the M1 marker *Nos2* (nitric oxide synthase/iNOS) and expression of a cytokine secreted by M1 macrophages *Tnfa* (tumor necrosis factor-α/TNFα, Fig. 3e), suggesting that the level of GM-CSF induced by miR-200c may be sufficient to enhance M1 antitumor macrophage polarization.

### Restoration of miR-200c in mammary carcinoma cells promotes M1 macrophage polarization via secreted factors

**Fig. 4:**
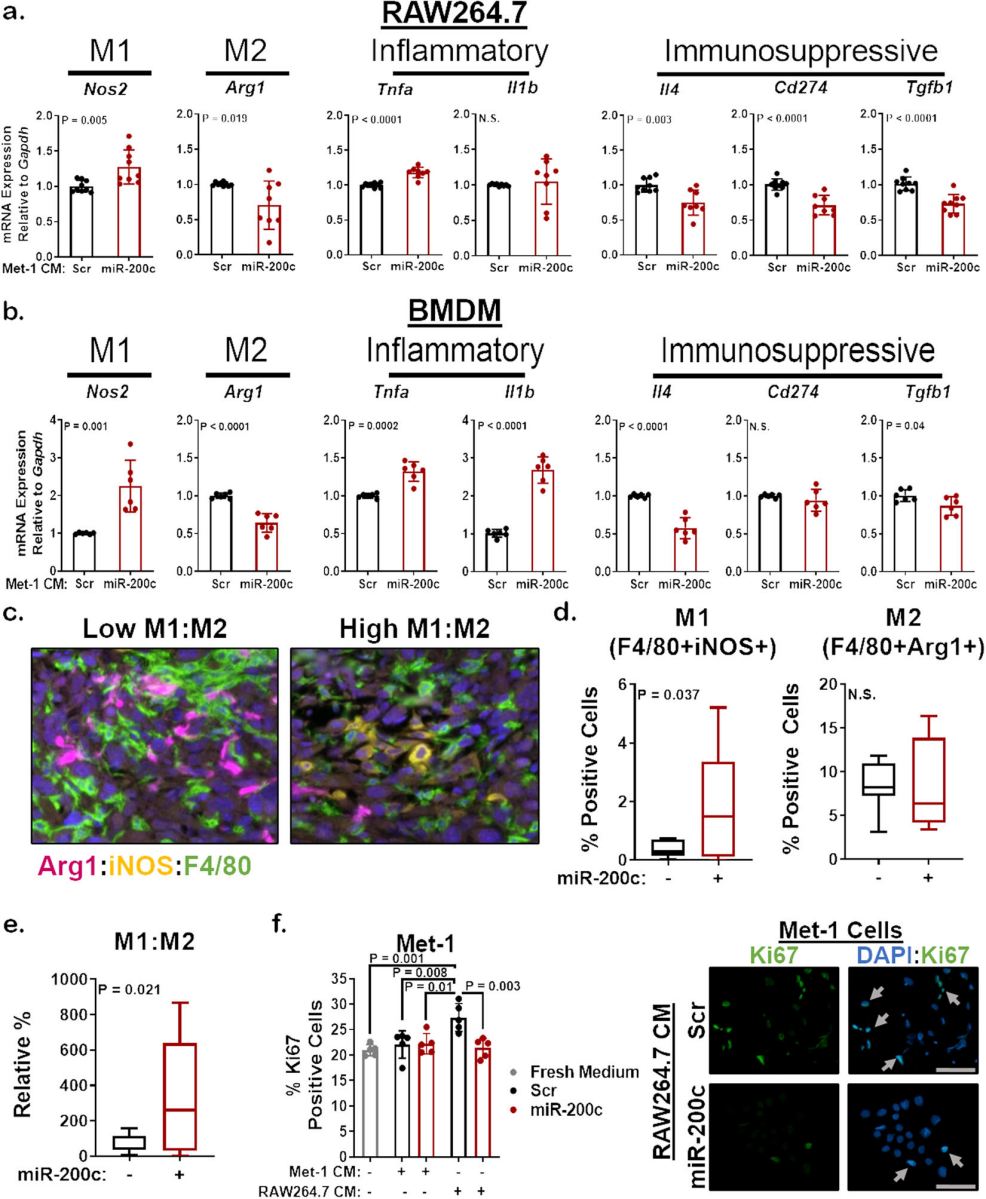
Soluble factors from miR-200c-expressing mammary tumor cells enhance M1 macrophage polarization. **a, b** Met-1 cells were transiently transfected with miR-200c. After 96 h, Met-1 Scr or Met-1 miR-200c conditioned medium (CM) was placed on RAW264.7 cells (**a**) or bone marrow-derived macrophages (BMDM) that were previously stimulated for 5 days with 25 ng/mL M-CSF (**b**). At 48 h culture with CM, macrophage polarization was determined via qRT-PCR for M1 (*Nos2* and proinflammatory cytokines: *Tnfa* and *Il1b*: interleukin-1 beta/IL-1β) and M2 (*Arg1*: Arginase-1/Arg1 and immunosuppressive cytokines/marker: *Il4*: Interleukin-4/IL-4, *Cd274*: programmed death-ligand 1/PD-L1, *Tgfb1*: transforming growth factor-β 1/TGF-β1) genes. Shown is the mean mRNA expression relative to *Gapdh* of 2–3 experiments conducted in triplicate ± s.d., *N* = 6–9, Student’s unpaired two-tailed *t* test. **c–e** M1 and M2 macrophage polarization was determined using opal multiplex flours on FFPE Met-1 TripZ-200c tumors. Images were scanned and analyzed using the Vectra Microscope and InForm Software. Representative fields from the same tumor with a low or high M1:M2 ratio are shown at 20× (magenta = Arg1, yellow = iNOS, green = F4/80, blue = DAPI, **c**). The percent M1 macrophages (F4/80+iNOS+ cells/total cells, **d**—left), percent M2 macrophages (F4/80+Arg1+ cells/total cells, **d**—right), and the ratio of M1:M2 macrophages (**e**) across 3–5 fields/tumor is shown as a box plot with the center line representing the median, the box representing the 25th and 75th percentiles and the whiskers representing the minimum and maximum, *N* = 7–11, Welsh’s unpaired two-tailed *t* test. **f** Shown is the mean percent Ki67-positive nuclei for Met-1 cells cultured with fresh culture medium (gray), CM from Met-1 cells transfected with miR-200c as described in (**a**) or CM from RAW264.7 cells that were polarized to more M2 or M1-like by CM from Met-1 Scr or miR-200c cells, respectively. Shown is the mean of five fields from 2–3 separate experiments ± s.d., *N* = 9–15, Student’s unpaired two-tailed *t* test (left). Representative images are shown on the right, 10×, scale bar = 100 µM (arrows denote Ki67-positive cells).

To determine whether miR-200c restoration promotes M1 antitumor macrophage polarization via secreted factors, such as enhanced GM-CSF, CXCL10, and CCL2, we transiently transfected Met-1 cells with Scr control or miR-200c mimic. At 96 h post transfection, we placed CM from tumor cells on to unactivated RAW264.7 cells and tested macrophage polarization via qRT-PCR. CM from Met-1 cells containing miR-200c significantly increased expression of the M1 marker *Nos2* and decreased expression of the M2 marker *Arg1* (arginase-1/Arg1) when compared to CM from Scr control containing cells (Fig. 4a). Other M1 markers, like the proinflammatory M1 cytokine *Tnfa* also significantly increased in macrophages treated with CM from miR-200c-containing Met-1 cells, while another proinflammatory cytokine *Il1b* showed variability in response to miR-200c-CM. Similar results were seen for the M1 markers *Nos2* and *Tnfa* after culturing RAW264.7 cells with CM from 66Cl-4 mammary carcinoma cells without or with miR-200c (Supplementary Fig. 6a). Since M2-like tumor associated macrophages (TAMs) are composed of several diverse and plastic populations^33^, we further characterized macrophages by their function, focusing on immunosuppression. Immunosuppressive markers like *Il4* (interleukin-4/IL-4), *Cd274* (PD-L1) and *Tgfb1* (transforming growth factor-β 1/TGF-β1) were significantly decreased by CM from miR-200c-transfected Met-1 cells (Fig. 4a).

Similar experiments were conducted with primary bone marrow-derived macrophages (BMDM). First, we stimulated BMDM with positive controls for M1 (IFNγ and LPS) and M2 (IL-4 and IL-13) macrophage polarization and all polarization markers tested increased in response to the appropriate positive control with the exception of *Cd274* (Supplementary Fig. 6b), demonstrating that most of the macrophage markers used in this study represent physiologically relevant M1 or M2 macrophage genes. BMDM were also cultured with CM from Scr control or miR-200c-containing Met-1 cells (Fig. 4b). MiR-200c-CM significantly increased gene expression of the M1 markers *Nos2*, *Tnfa*, and *Il1b* in BMDM when compared to CM from Met-1 Scr cells, while expression of the M2 markers *Arg1*, *Il4*, and *Tgfb1* significantly decreased. Therefore, secreted factors altered by miR-200c restoration in mouse mammary carcinoma cells enhanced inflammatory M1 macrophage markers and decreased immunosuppressive M2/TAM markers in both a macrophage cell line and primary macrophages.

We next analyzed macrophages in Met-1 TripZ-EV and Met-1 TripZ-200c tumors via IHC for F4/80 (a marker of all macrophages), Arg1, and iNOS (*Nos2*). Restoration of miR-200c in Met-1 TripZ-200c cells did not alter expression of F4/80 (Supplementary Fig. 7), suggesting that macrophage recruitment or proliferation were not altered after miR-200c restoration. However, the M2 marker Arg1 significantly decreased and the M1 marker iNOS increased, albeit nonsignificantly, in miR-200c expressing tumors, demonstrating altered macrophage polarization in vivo after restoring miR-200c (Supplementary Fig. 7). Since carcinoma cells as well as other cell types in the TME can express Arg1 or iNOS (reviewed in refs. ^34,35^), we confirmed these results using a custom fluorescent multiplex with antibodies specific for F4/80, Arg1, and iNOS to ensure that the changes in Arg1 and iNOS were macrophage specific. Macrophages were identified as F4/80+ cells and M1 or M2 macrophage polarization was determined by coexpression of iNOS or Arg1, respectively. The percent M1 antitumor macrophages significantly increased, while the percent M2 protumor macrophages trended toward a decrease in miR-200c-expressing tumors (Fig. 4c, d). Importantly, the ratio of M1:M2 macrophages significantly increased in miR-200c-expressing tumors (Fig. 4e), demonstrating that reverting mammary carcinoma cells to a more epithelial state enhanced M1 macrophage polarization markers in culture and in vivo.

Thus, far these studies demonstrate the impact of epithelial-like (miR-200c-positive) or mesenchymal-like (miR-200c-negative) BC cells on macrophages; however, depending on their polarization state, macrophages can in turn differentially impact BC cells. M1 macrophages decreased growth^36,37^ and increased death^38^ of BC cells, while M2 macrophages promoted BC cell EMT, proliferation, and migration^39^. Therefore, we examined this reciprocal relationship by testing the impact of miR-200c-induced M1 macrophage polarization on tumor cell proliferation. We cultured Met-1 cells with fresh growth medium or with CM from Met-1 cells without or with miR-200c restoration. Ki67 ICC showed no change in proliferation between these treatment groups (Fig. 4f, left panel), demonstrating that factors secreted by Met-1 Scr or Met-1 miR-200c cells did not impact proliferation. However, Met-1 cell proliferation significantly increased when cells were cultured with CM from RAW264.7 cells that were polarized to a more M2-like state following the same protocol as Fig. 4a (by CM from Met-1 Scr cells; Fig. 4f, left panel). Interestingly, this proliferative advantage was lost when Met-1 cells were cultured with CM from more M1-like RAW264.7 cells (polarized by CM from Met-1 miR-200c cells; Fig. 4f, right panel). These data demonstrate that factors secreted by M2-like macrophages, but not Met-1 cells or M1-like macrophages, support tumor cell proliferation via a reciprocal effect of macrophages on tumor cells.

### Cytokines altered by miR-200c predict enhanced M1 macrophage polarization and increased survival of TNBC patients

**Fig. 5:**
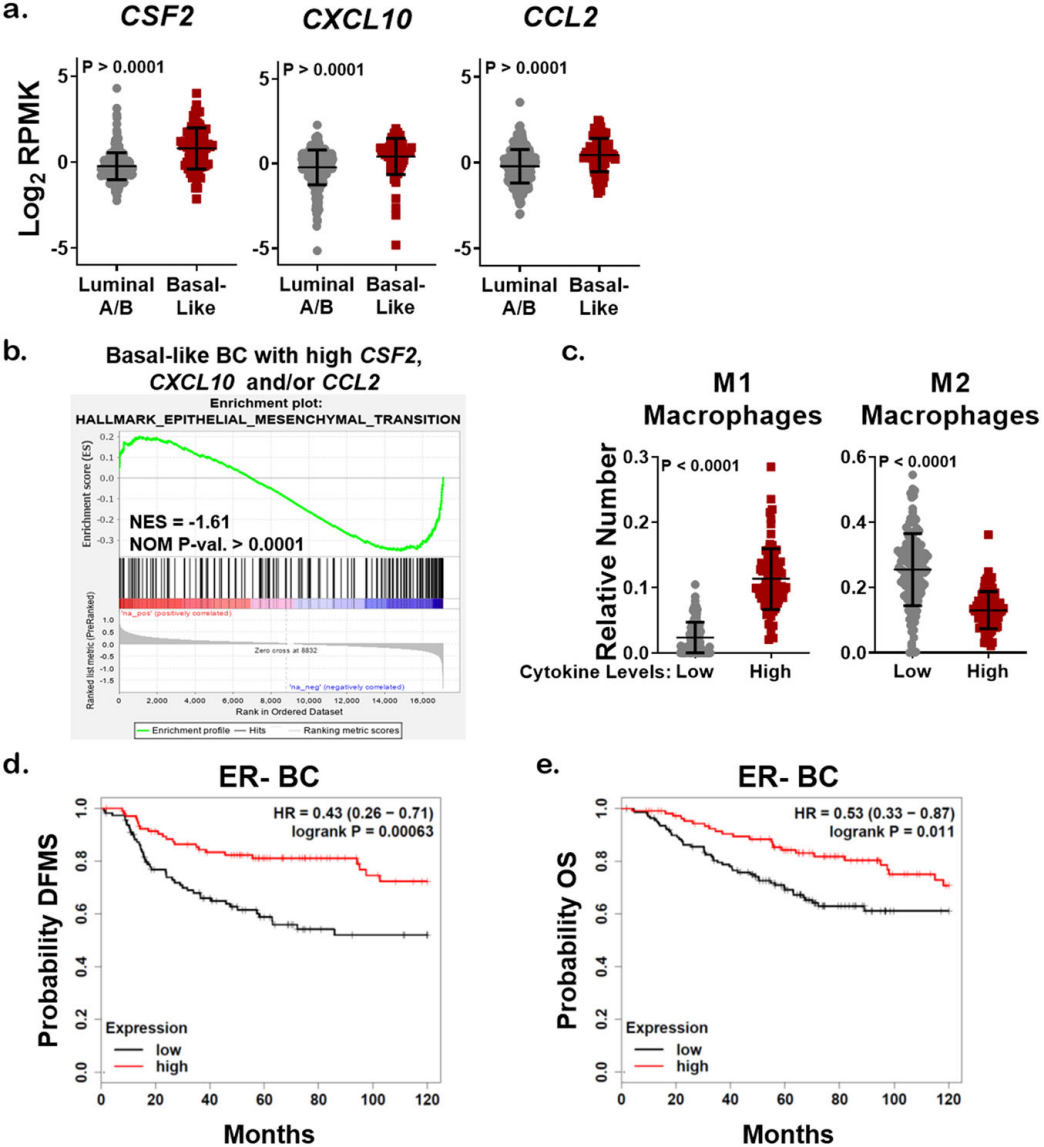
Cytokines upregulated by miR-200c restoration predict enhanced survival of TNBC patients. **a** mRNA expression of cytokines as curated by the Nature 2012 dataset in cBioPortal for luminal A/B (*N* = 321) or basal-like BC specimens (*N* = 81). Student’s unpaired two-tailed *t* test. **b** Genes enriched in basal-like BC specimens with expression of *CSF2*, *CXCL10*, and *CCL2* according to the dataset in **a** were subjected to GSEA analysis (Hallmark), normalized enrichment score (NES), nominal *p* value (NOM *p*-val). **c** mRNA profiling as curated by the Firehouse Legacy dataset (TCGA) was assessed for the relative number of immune infiltrates using CIBERSORT. Specimens with expression of *CSF2*, *CXCL10*, and/or *CCL2* in the lowest quartile (low, *N* = 92) or highest quartile (high, *N* = 132) were stratified via the relative number of M1 or M2 macrophages. Shown is the mean number of predicted immune cells ± s.d., Student’s unpaired two-tailed *t* test. KM plotter was used to stratify ER− BC patients with high expression of *CSF2*, *CXCL10*, and *CCL2* (red) based on distant metastasis-free survival (DMFS, **d**, *N* = 218) and overall survival (OS, **e**, *N* = 251).

To further assess the clinical relevance of our model, we explored alterations in the genes encoding GM-CSF, CXCL10, and CCL2 in BC patient cohorts using publicly available datasets. *CSF2*, *CXCL10*, and *CCL2* were commonly upregulated or amplified in claudin-low and basal-like BC (Supplementary Fig. 8a). When we stratified BC specimens using PAM50, a 50-gene signature that classifies BC into five molecular subtypes, basal-like tumors expressed more *CSF2*, *CXCL10*, and *CCL2* than luminal A/B tumors according to TCGA (Fig. 5a), a finding supported by previous studies^40,41^. Interestingly, mRNA expression of each cytokine varied widely between individual tumors regardless of subtype. Therefore, we interrogated the signaling pathways that were different between tumors with mRNA upregulation of *CSF2*, *CXCL10*, and *CCL2* using GSEA. The Hallmark-EMT pathway was one of the top pathways inversely correlated with high cytokine expression in both basal-like and luminal A/B BC (Fig. 5b and Supplementary Fig. 8b), suggesting that regardless of subtype, BC with high expression of one or more of these cytokines are more epithelial-like. BC in the TCGA cohort were analyzed by CIBERSORT that showed tumors with high expression of all three cytokines *CSF2*, *CXCL10*, and *CCL2* had significantly increased M1 macrophage polarization, decreased M2 macrophage polarization, and no change in the number of resting macrophages (M0) (Fig. 5c and Supplementary Fig. 8c). Finally, we stratified BC by high or low expression of all three cytokines, and high expression of *CSF2*, *CXCL10*, and *CCL2* predicted a better distant metastasis-free survival and overall survival (OS) for ER− BC patients (Fig. 5d, e). However, this was not the case for ER+ BC patients (Supplementary Fig. 8d, e), suggesting that TNBC patients with a high expression of miR-200c regulated cytokines may have an increased overall survival.

## Discussion

To understand mechanistically how BC EMT suppresses antitumor macrophages, we restored miR-200c to highly aggressive mesenchymal-like Met-1 mammary carcinoma and human TNBC cell lines. This work showed that miR-200c restoration and acquisition of a more epithelial state enhanced M1 antitumor macrophage polarization that was associated with induction of an immunogenic cytokine milieu. Interestingly, GM-CSF, CXCL10, and CCL2 correlated with a more epithelial state and enhanced M1 macrophage polarization in BC specimens. These cytokines only predicted a better OS in ER− BC patients, perhaps demonstrating the clinical significance of these findings for TNBC. Since EMT and the reciprocal MET are plastic processes and many BC cells exist in a state between fully epithelial or mesenchymal, we postulate that high GM-CSF, CXCL10, and CCL2 gene or protein expression may be used clinically to distinguish less aggressive TNBC with more epithelial features.

Although GM-CSF was associated with a more epithelial state in all models tested, the other miR-200c-induced cytokines were more variable. Intratumoral heterogeneity is a possible explanation for why our findings differ from a recent publication that observed enhanced neutrophil and decreased monocyte infiltration after miR-200c restoration^42^. In that study, miR-200c may have induced secretion of neutrophil-stimulatory cytokines; however, no analysis on protein expression of cytokines was conducted, warranting our study that delineated cytokines altered by miR-200c in additional models. Despite these limitations, the cytokines identified herein have well-established tumor suppressive roles and are being explored as anticancer agents in clinical trials with promising results.

GM-CSF is an important modulator of monocyte differentiation, maturation, and function. Single GM-CSF therapy decreased tumor progression in models of melanoma^43^ and BC^44^ by enhancing infiltration of M1 macrophages. EMT reversal in MMTV-PyMT tumors by SNAIL-depletion increased GM-CSF that induced M1 macrophage polarization and limited primary tumor growth and metastasis^29^. Since miR-200c directly targets *Snail* in addition to *Zeb1* and other mesenchymal transcription factors, it is likely that M1 macrophage polarization in miR-200c-expressing tumors and BC patients was mediated by enhanced GM-CSF expression. This idea is supported by our data showing that increasing GM-CSF concentrations enhanced M1 macrophage polarization markers. Further, M1 macrophages reciprocally affected BC cells by limiting tumor cell proliferation^36,37^ and increasing breast tumor cell death^38^. Consistent with these findings, our data demonstrated that more M2-like macrophages support a proliferative advantage in mammary carcinoma cells that is lost upon miR-200c-dependent M1 macrophage polarization. Therefore, aggressive TNBC may suppress GM-CSF to support a protumor paracrine loop between mesenchymal-like BC cells and M2 macrophages, suggesting that TNBC patients may benefit from GM-CSF therapy, which is currently being tested in other BC subtypes^45,46^. In advanced melanoma, GM-CSF therapies have been extensively studied where they enhanced dendritic cell antigen presentation and showed clinical benefit in combination with chemotherapy and checkpoint inhibitors^47^. Continued and expanded testing of GM-CSF should be considered in TNBC since M2 macrophage polarization predicts poor overall patient survival^48^ and M1 macrophage polarization predicts better survival (reviewed in ref. ^49^).

CXCL10 is a chemoattractant for T cells, natural killer cells, and monocytes. In the context of cancer immunotherapy, CXCL10 enhanced dendritic cell-antigen presentation and subsequent tumor cell killing by T cells in the presence of tumor vaccines in melanoma^50,51^ and glioma models^52,53^. CXCL10 is being explored in combination with a glioma tumor vaccine (NCT02549833). Although testing of CXCL10 as an immunostimulant in BC has not begun, it was highly expressed in 45% of primary BC regardless of subtype where CXCL10 correlates with increased CD4 and CD8 Tcell infiltration^54^. Since CXCL10 is used to activate dendritic cell antigen presentation clinically, it is possible that CD8 T cell activation is also enhanced after miR-200c restoration. This idea is supported by our findings that mRNA encoding the checkpoint protein PD-L1 was decreased in macrophages after exposure to CM from miR-200c-containing mammary carcinoma cells. Although EMT reversal limited tumor cell checkpoint protein expression^16–18^ and induced tumor cell antigen presentation^8^, the impact of BC EMT reversal on macrophage and dendritic cells and its subsequent impact on T cell cytotoxicity needs to be further explored.

CCL2 has an established role in recruiting inflammatory macrophages into breast tumors to increase primary tumor growth^55,56^ and metastasis^57^. Although CCL2 similarly supports metastasis in preclinical studies of other cancers^58^, targeting CCL2 had no antitumor activity in castrate resistant prostate cancer^59^. These discrepancies may be explained by the fact that CCL2 recruits monocytes that then differentiate to pro- or antitumor macrophages depending on the cytokine milieu of each tumor (reviewed in ref. ^60^). In the context of miR-200c, CCL2 may recruit monocytes that are polarized to M1 macrophages by cosecretion of GM-CSF. Therefore, upregulation of GM-CSF, CXCL10, and CCL2 in response to miR-200c restoration may break a vicious paracrine loop between BC cells and macrophages known to support tumor progression^61^. In future studies, we will test the idea that enhanced expression of GM-CSF, CXCL10, and CCL2 is needed in tandem to elicit a potent antitumor immune response.

Profiling of BC metastases in comparison to matching primary tumors has shown that metastases, regardless of BC subtype or metastatic site, have increased monocyte/M2 macrophage infiltration^62,63^. However, all other immune cells were found to be decreased in metastases compared to primary tumors. Similarly, single-cell profiling of a metastatic TNBC patient who did not respond to combined chemotherapy and checkpoint inhibition demonstrated that myeloid cell populations, including M2 macrophages, were enriched prior to and sustained throughout treatment^64^. These studies highlight the need to develop clinical strategies to revert protumor M2 macrophages into antitumor M1 macrophages, particularly in the metastatic setting. Recent development of small molecules and other therapies that restore miR-200c expression in TNBC cells could be viable approaches to activate innate immune cells against aggressive mesenchymal-like tumors^65,66^. These therapies or tumor-specific delivery of GM-CSF, CXCL10, and CCL2 could be successful with chemotherapy or in combination with checkpoint inhibitors.

The study presented herein utilized miR-200c as a tool to identify an immunogenic cytokine milieu that can indicate EMT status and prognosis of TNBC patients. For aggressive mesenchymal-like TNBC, GM-CSF alone or with CXCL10 and CCL2 may activate both innate and adaptive antitumor immune responses that could combine favorably with current treatment approaches such as chemotherapy and checkpoint inhibition.

## Methods

### Cell culture conditions

Met-1 (RRID:CVCL_U373), Met-1 TripZ-EV, Met-1 TripZ-200c, and RAW264.7 (RRID:CVCL_0493) cell lines were maintained in DMEM with 10% FBS and 1% pen/strep. 66Cl-4 (RRID:CVCL_9721) cells were cultured in DMEM with 10% FCS supplemented with 1% NEAA, L-glutamine, and pen/strep. BT549 (ATCC Cat# HTB-122, RRID:CVCL_1092) and BT549 TripZ-200c cells were maintained in RPMI-1640 with 10% FBS, 1% NEAA and pen/strep, and 5 µg/mL insulin. SUM159PT (RRID:CVCL_5423) cells were cultured in Ham’s F12 supplemented with 5% FCS, 1% HEPES, 0.5% pen/strep, 5 µg/mL insulin, and 3.2 ng/mL hydrocortisone. All cells were utilized for experiments within eight or fewer passages. Mouse mammary carcinoma cells were kindly provided by Drs Donald McDonnell and Alexander Borowsky (cells were generated as described^25^). RAW264.7 cells were kindly provided by Dr Phillip Owens. Human BC cells were purchased from the ATCC (BT549 in 2008) or from the University of Colorado Anschutz Medical Campus (CU AMC) Cell Culture Services Core (SUM159PT in 2013). Cells were short tandem repeat fingerprinted by the CU AMC Cell Culture Services Core and had 100% match to ATCC for BT549 cells and Asterand for SUM159PT cells (August 2017). All cells were tested for mycoplasma contamination at least every 3 months using the MycoAlert PLUS detection kit (Lonza Cat# 75860-358).

### Inducible miR-200c model

An inducible lentiviral pTripZ-RFP vector (Dharmacon Cat# RHS4750), kindly provided by the lab of Dr Rui Yi, encoding an empty vector or precursor sequence for miR-200c (TripZ-EV or TripZ-200c) was transduced into Met-1 cells. Met-1 TripZ-EV cells were a pool of multiple clones, while Met-1 TripZ-200c cells represent a single clone. The pTripZ vector has an RFP tag and Met-1 TripZ-EV (pool of clones) and Met-1 TripZ-200c (single clone) cells each went through negative selection via flow cytometry to generate nonleaky cell lines. To select for cells that induced intermediate expression of miR-200c, only the Met-1 TripZ-200c cells went through a second round of selection for cells with intermediate RFP-positivity after treatment with 1.0 µg/mL Dox (Sigma Cat# D9891). For all cell culture experiments, Met-1 TripZ-EV or Met-1 TripZ-200c cells were similarly treated ± 1.0 µg/mL Dox.

### Transient miR-200c model

Mammary carcinoma cells were plated in six-well plates (100,000–200,000 cells/well). The following day cells were transfected with 40 nM Scr control or miR-200c mimic (Thermo Fisher, Cat# 4464058 and Cat# 4464066, respectively). At 16 h post transfection, medium containing transfection reagents was removed and replaced with fresh appropriate medium.

### Quantitative real-time PCR

Total mRNA was isolated using TRIzol (Thermo Fisher Cat# 15596018) extraction according to the manufacture’s suggestions. One microgram of total mRNA was reverse transcribed into cDNA using QScript (Quantabio Cat# 95048-025) according to the manufacture’s protocol. Expression (∆∆CT) of genes of interest were determined relative to *Gapdh* by qRT-PCR using PowerUP SYBR Green Master Mix (Thermo Fisher Cat# A25742), a 7500 Fast Real-Time PCR System (Applied Biosystems), and 7500 Software (ver2.3, RRID:SCR_014596). The exception was *miR-200c* that was detected using TaqMan probes and was presented relative to expression of *U6*. Primers were purchased, designed, and/or selected from the Harvard Primer Bank database^67^ and are shown in Supplementary Data 7.

### Western blot analysis

Cells were lysed in radioimmunoprecipitation buffer supplemented with 1x Halt protease and phosphatase inhibitor (Thermo Fisher Cat# 78442) and cell debris was removed by centrifugation. Protein concentration was determined using a Pierce BCA Protein Assay Kit (Thermo Fisher Cat# 23225) according to the manufacture’s protocol. 35–50 µg protein was run on a 10% SDS-PAGE gel that was transferred to a methanol activated PVDF membrane. Protein expression was determined using the Odyssey CLx imaging system (RRID:SCR_014579) and Image Studio Software (ver5.2, RRID:SCR_015795) after overnight incubation with primary antibodies at 4 °C (β-Actin 1:10,000, clone# 13E5, lot# 15, Cell Signaling Technology Cat# 4970, RRID:AB_2223172; CDH1 1:1000, clone# 24E10, lot# 12, Cell Signaling Technology Cat# 3195, RRID:AB_2291471; ZEB1 1:1000, lot# G115263, Sigma-Aldrich Cat# HPA027524, RRID:AB_1844977) and a 1 h incubation at room temperature with secondary antibody (Goat anti-Rabbit Secondary 1:5000, lot# C90619-05, LI-COR Biosciences Cat# 925-32211, RRID:AB_2651127). All westerns presented in a single figure panel are from the same experiment and full uncropped images can be seen in Supplementary Fig. 9.

### Immunocytochemistry

Met-1 cells were plated in six-well dishes containing cover slips (25,000–50,000 cells/well). After 96 h Dox treatment (1.0 µg/mL) or 48 h treatment with CM, cells were fixed in 70% acetone/30% methanol, blocked in 10% Normal Goat Serum, and incubated with primary antibody overnight at 4 °C (CDH1 1:400, clone# 24E10, lot# 12, Cell Signaling Technology Cat# 3195, RRID:AB_2291471; Ki67 1:500 Abcam Cat# ab15580, RRID:AB_443209), and then incubated with secondary antibody for 2–3 h at room temperature (anti-Rabbit Secondary 1:100,lot# 1705869 Thermo Fisher, A-11008). Cells were stained with DAPI prior to imaging on a BX40 microscope (Olympus) with a SPOT Insight Mosaic 4.2 camera (Diagnostic Instruments) and cellSens Standard Software (ver2.3, RRID:SCR_014551). Analysis of percent Ki67-positive cells was conducted using ImageJ Software (RRID:SCR_003070) on five fields (10X each) from two to three separate experiments.

### Cell proliferation

Met-1 TripZ-EV and Met-1 TripZ-200c cells were plated in 96-well plates (500 cells/well) and the following day cells were treated ± 1.0 µg/mL Dox. After 6 days treatment, cells were stained with 5% crystal violet and cell viability was determined by solubilizing in 5% citrate buffer and measuring optical density at 540 nm using a Synergy 2 (Biotek) plate reader and Gen5 Software (ver2.00, RRID:SCR_017317).

### Growth in soft agar

Met-1 TripZ-EV and Met-1 TripZ-200c cells were plated in six-well plates (20,000 cells/well) containing 0.5% bottom agar, 0.3% top agar with cells, and covered with the appropriate medium ± 1.0 µg/mL Dox. Cells were cultured for 14 days changing the medium and adding fresh Dox biweekly. Cells were fixed and stained with nitro blue tetrazolium and colony number was quantified using ImageJ Software (RRID:SCR_003070).

### Migration and invasion

Met-1 TripZ-EV and Met-1 TripZ-200c cells were plated into 96-well ImageLock Plates (Essen Bio, Cat# 4379, 50,000 cells/well) and the following day were wounded with the IncuCyte WoundMaker Tool (Essen Bio, Cat# 4493) and treated ± 1.0 µg/mL Dox. Plates were scanned every 4 h post wounding with the IncuCyte Zoom system (RRID:SCR_019874) and percent wound closure was quantified using IncuCyte Zoom Software (ver2018A). For invasion assays 100 µL, reduced growth factor basement membrane (Cultrex, Cat# 3433-005-01) was plated on top of cells and within the wound following wounding.

### Animal experiments

All animal experiments were performed in accordance with a protocol (#00407) approved by the University of Colorado Institutional Animal Care and Use Committee using humane procedures. Met-1 TripZ-EV or Met-1 TripZ-200c cells (200,000 cells/tumor) were injected bilaterally into the fourth inguinal mammary fat pads of 6–8-week-old female FVB/NJ (IMSR Cat# JAX:001800, RRID:IMSR_JAX:001800) syngeneic hosts (*N* = 10 mice/group). Mice were monitored for tumor growth every other day. When the tumor volume, determined by caliper measurements (volume = 1/2(width^2^ × height)), reached an average of 50 mm^3^ mice were randomized to treatment with Dox delivered in the chow (3.0 mg/kg/mouse daily, Envigo Cat# TD.01306) or standard chow control (*N* = 5 mice/group). When the average tumor volume for each group reached 500 mm^3^, mice were sacrificed and half of each tumor was FFPE while the other half was snap frozen for gene analysis. Mice that developed ulcers were excluded from the study. The data shown are an average of two separate experiments.

### Cytokine analysis

CM from Met-1 TripZ-200c or BT549 TripZ-200c cells treated ± 1.0 µg/mL Dox for 72 h was frozen at −80 °C. Cytokines in the mouse model were profiled following the instructions in the Proteome Profiler Mouse Cytokine Array Panel A kit (R&D Cat# ARY006), while the BT549 CM was analyzed using the Meso Scale Discovery 30 Cytokine V-PLEX Panel (MSD Cat# K15054D-1). Protein expression of GM-CSF was confirmed in CM using the LEGEND MAX Mouse GM-CSF ELISA kit (Biolegend Cat# 432204) that was read on a Synergy 2 (Biotek) plate reader using Gen5 Software (ver2.00, RRID:SCR_017317).

### Macrophage polarization

At 96 h transient miR-200c restoration, 1.5 mL CM from cancer cells and 500 µL fresh growth medium were placed onto RAW264.7 macrophages that were 50% confluent. At this time mRNA was extracted from mammary carcinoma cell lines to confirm miR-200c transfection and cytokine gene expression (as described above under “Quantitative real-time PCR”). After 48 h treatment with CM, mRNA was extracted from macrophages using TRIzol and macrophage polarization was assessed via qRT-PCR (as described above under “Quantitative real-time PCR”). For experiments with primary BMDM, bone marrow was isolated from the hind tibia and femur of two wild-type FVB/NJ mice as described in ref. ^68^. Briefly, the hind tibias and femurs were centrifuged after removal of connective and soft tissue to collect the bone marrow. Isolated cells were plated in 10 cm petri dishes (125,000 cells/plate) and primed for M2 polarization with 25 ng/mL M-CSF or M1 macrophage polarization with 5 ng/mL GM-CSF. After 5 days M-CSF or GM-CSF treatment, 5 mL of medium was removed from each BMDM plate and replaced with 5 mL fresh Met-1 growth medium containing 20 ng/mL IL-4 and 20 ng/mL IL-13 or 20 ng/mL IFNγ and 100 ng/mL LPS to promote M2 and M1 macrophage polarization, respectively. Additional BMDM were similarly treated with CM from Met-1 Scr or Met-1 miR-200c cells and qRT-PCR (as described above under “Quantitative real-time PCR”) was used to assess macrophage polarization.

### Tumor cell proliferation in response to macrophage polarization

Met-1 cells were plated on cover slips in six-well dishes (25,000 cells/well) and treated with 2.0 mL fresh growth medium or 1.0 mL fresh growth medium plus 1.0 mL of Met-1 Scr CM, Met-1 miR-200c CM, M2-like RAW264.7 CM (polarized by CM from Met-1 Scr cells as described above under “Macrophage polarization”), or M1-like RAW264.7 CM (polarized by CM from Met-1 miR-200c cells as described above under “Macrophage polarization”). Met-1 cell proliferation was determined after 48 h treatment with CM by ICC for Ki67 that was conducted as described above under “Immunocytochemistry.”

### Immunohistochemistry

Cell pellets generated from Met-1 TripZ-EV and Met-1 TripZ-200c cells or FFPE tissues from animal studies were analyzed via hematoxylin & eosin staining or IHC for: CDH1 (1:400, clone# 24E10, lot# 12, Cell Signaling Technology Cat# 3195, RRID:AB_2291471), PyMT (1:200 Novus Cat# NB100-2749, RRID:AB_10001944), F4/80 (1:400, clone# BM8, lot# 1711898, Thermo Fisher Scientific Cat# MF48000, RRID:AB_10376289), Arg1 (1:200, clone# D4E3M, lot# 1, Cell Signaling Technology Cat# 93668, RRID:AB_2800207), iNOS (1:100, clone# D6B6S, lot# 5, Cell Signaling Technology Cat# 13120, RRID:AB_2687529), and CD8 (1:800, clone# 4SM15, lot# 4348256, Thermo Fisher Scientific Cat# 14-0808-80, RRID:AB_2572860). Antigen retrieval was optimized for each antibody that was detected using either the ImmPRESS goat anti-rabbit HRP polymer (Vector Laboratories Cat# MP-7451, RRID:AB_2631198) or ImmPRESS goat anti-rat HRP polymer (Vector Laboratories Cat# MP-7444, RRID:AB_2336530) and ImmPACT DAB substrate (Vector Laboratories Cat# SK-4105, RRID:AB_2336520). All IHC was analyzed by scanning the entire slide with the Aperio microscope and analyzing the entire tumor section using the ImageScope x64 Software (ver12.4.0, RRID:SCR_014311). Data shown are the mean percent positive pixels.

### Multispectral fluorescence

The multiplex panel was designed using multiplex Opal™ TSA technology (Akoya Biosciences) using sequential rounds of antibody probing/detecting and heat retrieval. Slides were imaged with the Vectra 3 Automated Quantitative Pathology Imaging System. Antibodies used were F4/80 (1:6400 clone# BM8, lot# 1711898, Thermo Fisher Scientific Cat# MF48000, RRID:AB_10376289), Arg1 (1:800, clone# D4E3M, lot# 1, Cell Signaling Technology Cat# 93668, RRID:AB_2800207), and iNOS (1:200, clone# D6B6S, lot# 5, Cell Signaling Technology Cat# 13120, RRID:AB_2687529) that were detected with the either the ImmPRESS goat anti-rabbit HRP polymer (Vector Laboratories Cat# MP-7451, RRID:AB_2631198) or ImmPRESS goat anti-rat HRP polymer (Vector Laboratories Cat# MP-7444, RRID:AB_2336530) and Opal flours 520 (Akoya Biosciences Cat# FP1487001KT), 570 (Akoya Biosciences Cat# FP1488001KT), and 690 (Akoya Biosciences Cat#FP1497001KT). DAPI (Akoya Cat# FP1490) was used as a counterstain. From each tumor, macrophages in 3–5 669 µm × 500 µm fields were scored for percent positive M1 (F4/80+iNOS+ cells/total cells) or M2 (F4/80+Arg1+ cells/total cells) cells using Inform Software (Perkin Elmer, ver2.4, RRID:SCR_019155).

### Bioinformatics and analysis of publically available datasets

TRIzol was used to extract total mRNA from Met-1 TripZ-200c cells treated ± Dox for 48 h as described above. Total mRNA was prepared for bulk mRNA sequencing completed using the TruSeq Stranded RNA kit according to the manufacturer’s instructions to generate Illumina HiSeq libraries that were sequenced as single pass 50 bp reads with the Illumina HiSeq4000 platform (RRID:SCR_016386). Sequencing was completed in the CU AMC Genomics Shared Resource and the resulting data were analyzed via a custom pipeline used previously^69,70^. Briefly, this pipeline was composed of the following steps: open-source gSNAP (RRID:SCR_005483) was used to map reads to the mouse genome (GRCM38.p6 FVB_NJ_v1)^71^, Cufflinks (RRID:SCR_014597) was used to determine expression (FPMK)^72^, and R (RRID:SCR_001905) was used for sequence alignment and to determine differentially expressed genes using ANOVA. Differentially expressed genes (*Q* < 0.05) were used for downstream analyses and these data were deposited in NCBI’s Gene Expression Omnibus^73^ (RRID:SCR_005012) and are accessible through GEO Series accession number GSE151320. GO analysis was determined by assessing the top genes upregulated and downregulated by miR-200c using DAVID 6.8 (GOTERM_BP_DIRECT, RRID:SCR_001881)^74,75^. Preranked GSEA (ver4.0.3, RRID:SCR_003199)^76,77^ was similarly conducted on combined top upregulated and downregulated genes differentially expressed between cells without and with miR-200c restoration using the Hallmark Gene Sets (Broad Institute). The top 50 Hallmark-EMT genes altered by Dox treatment in Met-1 TripZ-200c cells were presented in a heatmap. Genes altered by restoration of miR-200c were compared to published datasets^15,16^ (GEO accession numbers GSE62230 and GSE108271, respectively) by inputting top genes downregulated by miR-200c in each dataset into BioVenn^78^. TCGA^79^ and METABRIC^80^ datasets were mined using cBioPortal^81,82^ and a *z*-score threshold of 1.5 to observe alterations in genes, expression of genes, and coexpressed genes across BC subtypes. Preranked GSEA was also conducted on this TCGA dataset using the Hallmark Gene Sets (Broad Institute) on genes enriched in basal-like or luminal A/B BC specimens with expression of *CSF2*, *CXCL10*, and *CCL2*. Relative immune cell composition of BC specimens in the TCGA Firehouse Legacy dataset was determined using CIBERSORT^83^, a publically available computational method to quantify immune cells from bulk sequencing data. Briefly, gene expression data were uploaded to the CIBERSORT online portal and compared it to gene signatures from 22 functionally defined human immune cell types (LM22). CIBERSORT is presented for patients with *CSF2*, *CXCL10*, and *CCL2* in the top 25% (high) or bottom 25% (low). Survival analysis was conducted using Kaplan–Meier plotter (KM plotter^84^) on patients with either ER− or ER+ disease using the mean expression of all jet set probes for *CSF2*, *CXCL10*, and *CCL2* and the best cut-off.

### Statistical analysis

All tissue culture experiments are the mean of three separate experiments conducted in triplicate to decuple and values two standard deviations above or below the mean were excluded for any experiment with an *N* ≥ 9. When appropriate significance was determined using a Student’s or Welsh’s unpaired two-tailed *t* test or an ANOVA with a Tukey’s multiple comparison test. Animal numbers were calculated at 80% power to the expected difference (based on published studies^15^) at *p* < 0.05 (two-tailed). Significance for tumor volume measurements was calculated using a repeated measures two-way ANOVA. All statistics were calculated using GraphPad Prism software (ver8, GraphPad, La Jolla, CA, RRID:SCR_002798) and were defined as significant if *p* < 0.05; however, *p* value < 0.1 was also reported to demonstrate a trend toward significance.

### Reporting summary

Further information on research design is available in the Nature Research Reporting Summary linked to this article.

## Supplementary information

Supplementary Information

Supplementary Data 1

Supplementary Data 2

Supplementary Data 3

Supplementary Data 4

Supplementary Data 5

Supplementary Data 6

Supplementary Data 7

Reporting Summary

## Data Availability

For any novel reagent or resource (e.g., Met-1 TripZ-EV or Met-1 TripZ-200c cell lines) created in the course of this project, we will follow the NIH policy on timely distribution and sharing on biomedical research resources, as published in the NIH Grants Policy Statement. As appropriate, sharing will be under the guidance of the CU Innovations technology transfer operations. mRNA sequencing data shown in Fig. 1e–g, Supplementary Fig. 1b, c, and Supplementary Data 1–6 are already deposited in NCBI’s Gene Expression Omnibus^73^ and are accessible through GEO Series accession number GSE151320. Additional data files generated and analyzed during this study are described and shared in the following *figshare* data record: 10.6084/m9.figshare.14456310^85^.
