# Supplementary material for: MicroRNA-200c restoration reveals a cytokine profile to enhance M1 macrophage polarization in breast cancer
Source: NPJ Breast Cancer. 2021 May 27;7:64. doi: 10.1038/s41523-021-00273-1 (PMC8160264; doi:10.1038/s41523-021-00273-1)

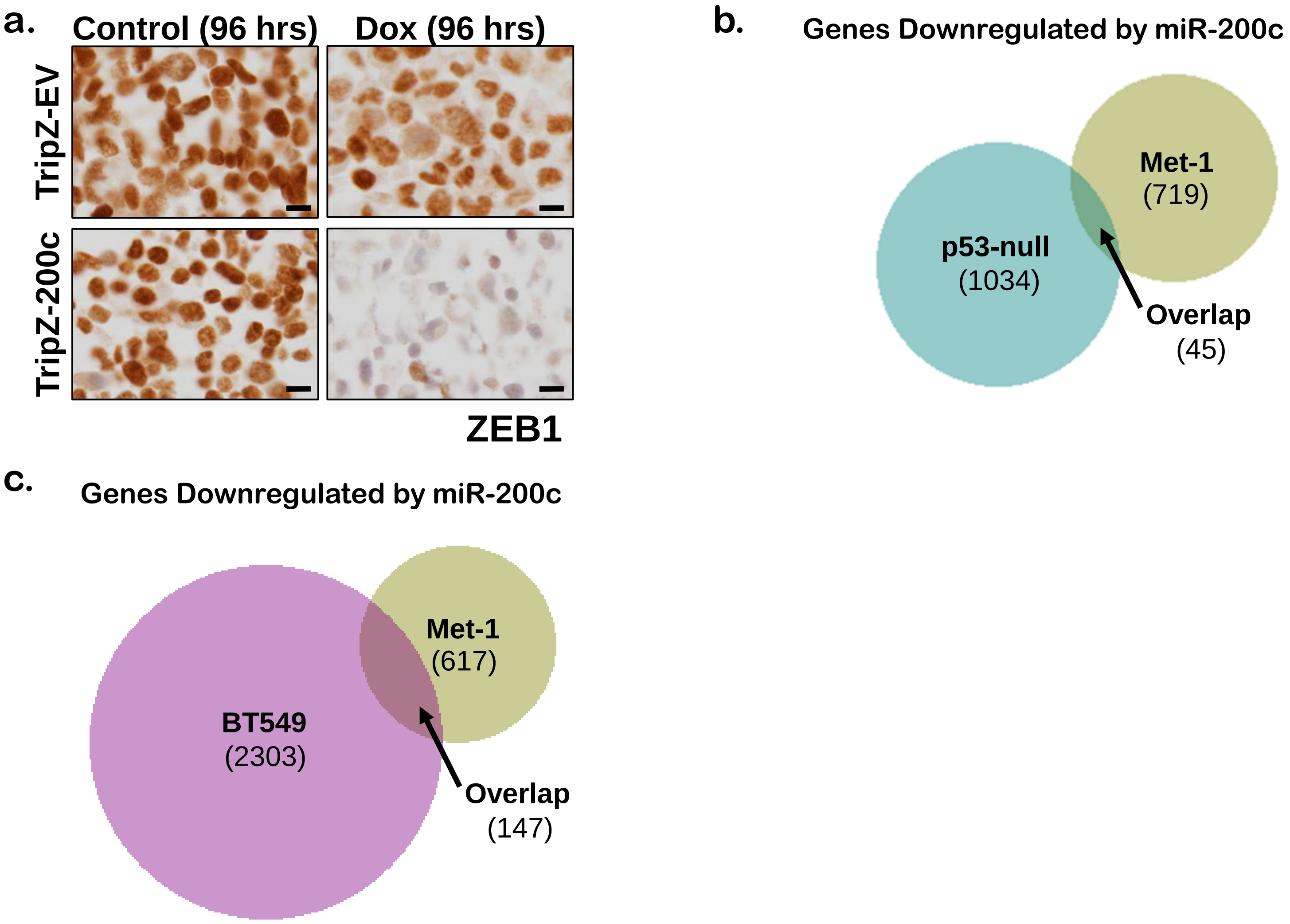

Supplementary Figure 1. a. Met-1 TripZ-EV and Met-1 TripZ-200c cells were treated with 1.0 µg/mL Dox for 96 hours and cells were pelleted and FFPE. Shown are representative images for staining of ZEB1, 40x, scale bar = 10 µM. **b-c.** Genes downregulated by miR-200c in Met-1 TripZ-200c cells were compared to genes downregulated by miR-200c in a p53-null claudin-low mammary tumor model (**b**) or genes downregulated by miR-200c in human TNBC BT549 cells (**c**). Gene lists can be seen in Supplementary Tables 5 and 6.

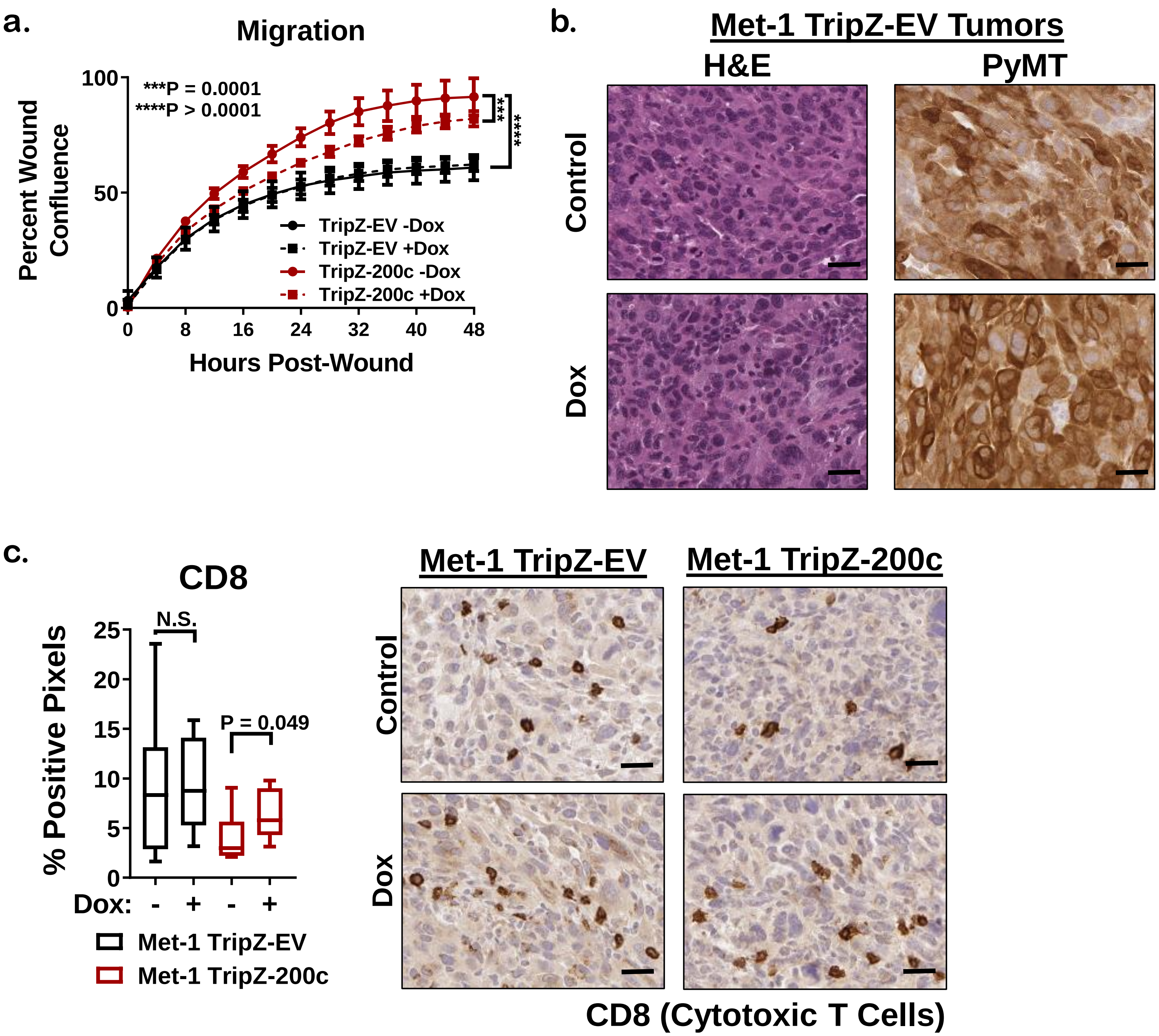

Supplementary Figure 2. a. Migration of Met-1 TripZ-EV and Met-1 TripZ-200c cells treated with 1.0 µg/mL Dox for 48 hours was determined using the IncuCyte imaging system, shown is the mean percent wound confluence for two experiments ± s.d., N = 20, two-way ANOVA. **b.** Met-1 TripZ-EV tumors were analyzed by staining for Hematoxylin & Eosin (H&E) and PyMT. Shown is a representative image for each group, 10x, scale bar = 30 µm. **c**. IHC for CD8 was conducted on Met-1 TripZ-EV and Met-1 TripZ-200c tumors. Quantification was done on the entirety of each tumor using the Aperio microscope and ImageScope software. Percent pixels are presented as a box plot with the center line representing the median, the box representing the 25th and 75th percentiles and the whiskers representing the minimum and maximum, N = 8-12, Student's unpaired two-tailed T-test (left). Representative images are shown (right), 10x, scale bar = 30 µm.

a.

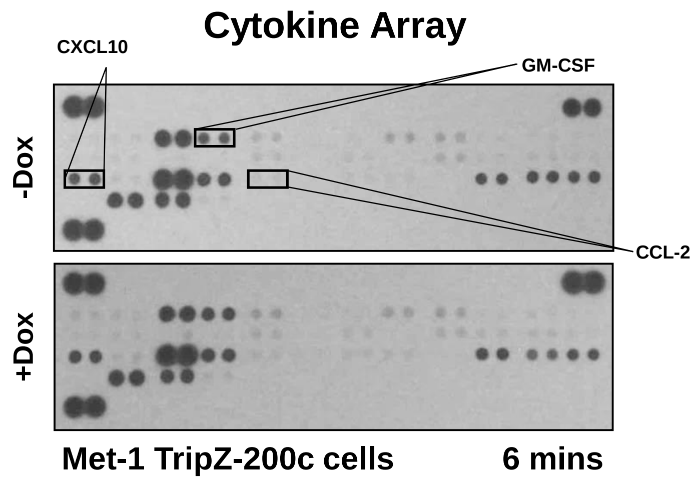

b.

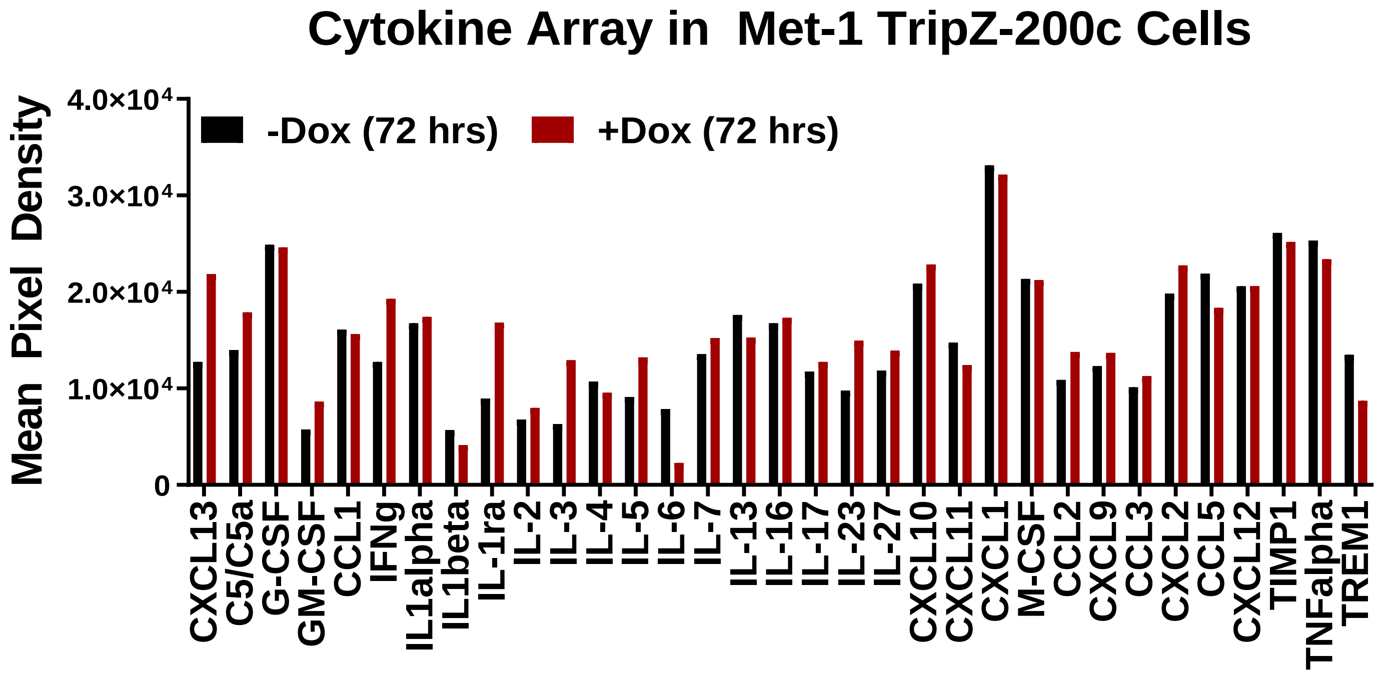

Supplementary Figure 3. a-b. Met-1 TripZ-200c cells were treated ± 1.0 µg/mL Dox for 72 hours to restore miR-200c. At this time point, conditioned medium from cells was harvested and analyzed via cytokine array. A representative image for a 6 minute exposure is shown (**a**). Quantification of mean pixel density for all cytokines detected was completed using ImageJ and is shown (**b**).

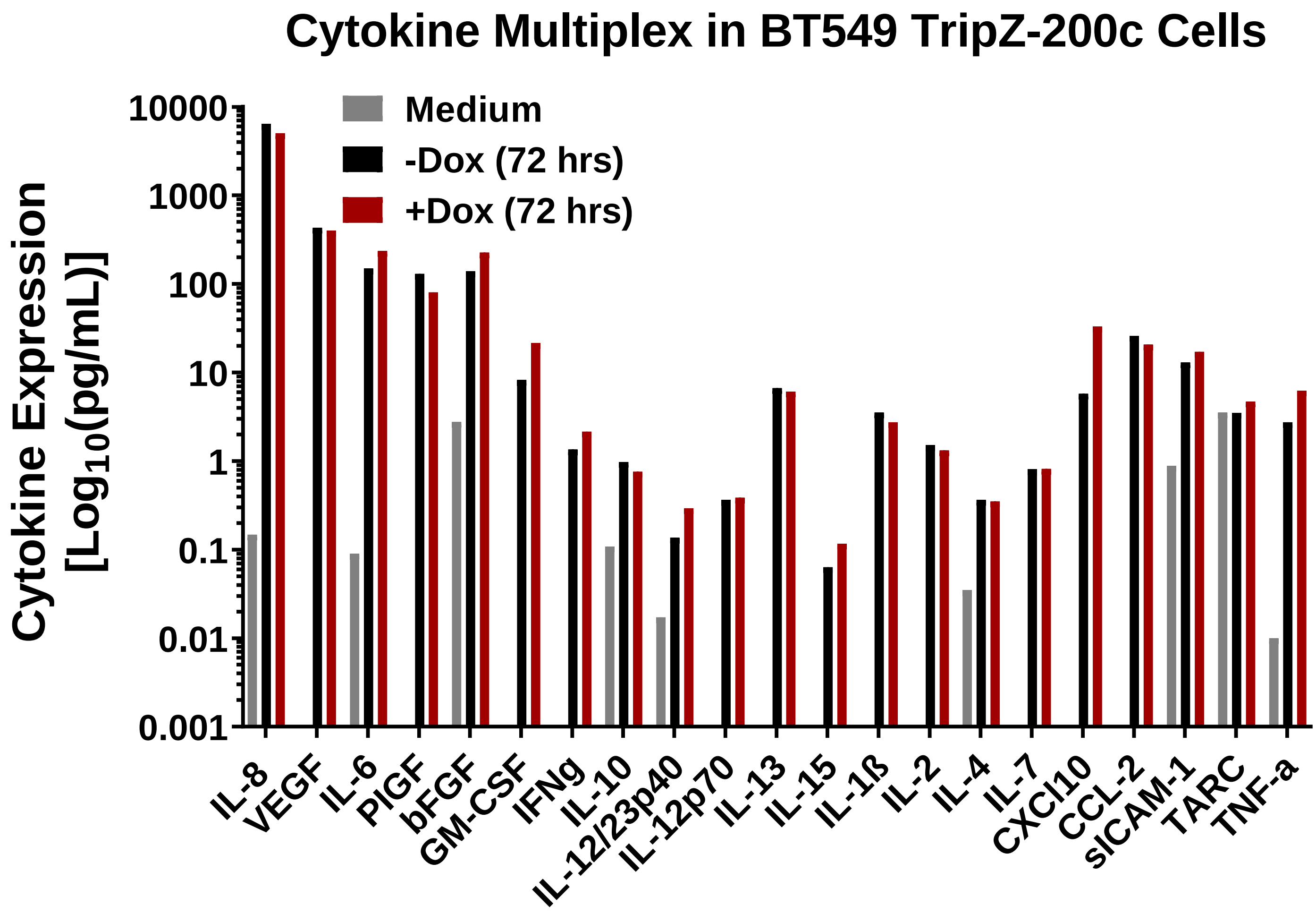

Supplementary Figure 4. BT549 TripZ-200c cells were treated ± 1.0 µg/mL Dox for 72 hours to restore miR-200c. At this time point, conditioned medium from cells was harvested and analyzed via cytokine multiplex. Shown is the cytokine expression for all cytokines detected above baseline expression in medium alone (grey).

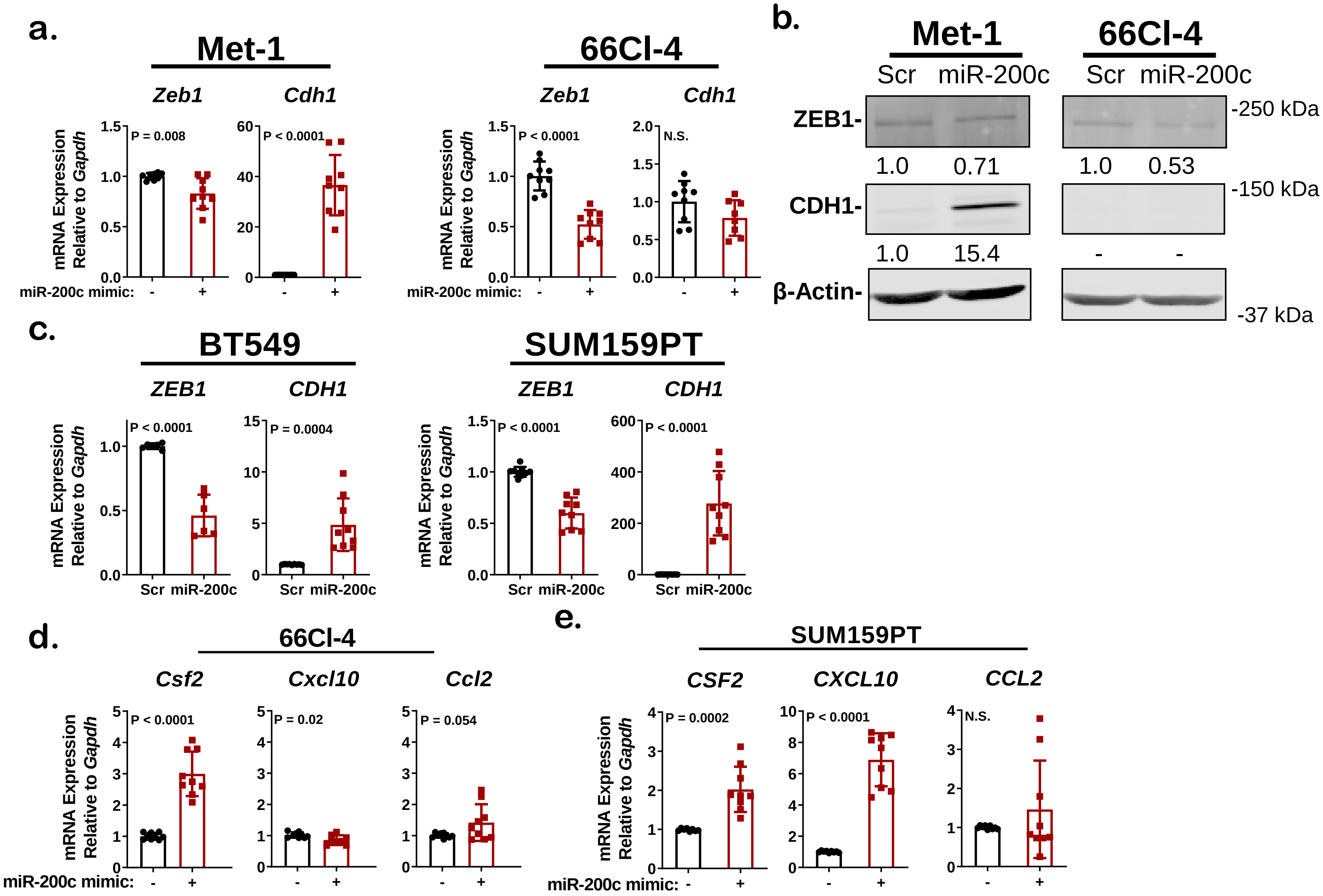

Supplementary Figure 5. a-b. Restoration of miR-200c was confirmed in Met-1 and 66Cl-4 mammary carcinoma cells after 96 hours transient transfection with Scramble control (Scr) or miR-200c mimic (miR-200c) by qRT-PCR (**a**) and western blot analysis (**b**) for ZEB1 and CDH1**.** Densitometry was conducted using ImageJ and expression of ZEB1 and CDH1 was normalized to β-Actin. **c.** Restoration of miR-200c was confirmed in human TNBC BT549 and SUM159PT cells after 48 and 96 hours transient transfection, respectively, with Scr or miR-200c via qRT-PCR for *ZEB1* and *CDH1*. **d-e.** mRNA expression of cytokines of interest were determined by qRT-PCR in 66Cl-4 (**d**) and SUM159PT (**e**) cells after transient miR-200c restoration for 96 hours. All qRT-PCR data shown is the mean mRNA expression relative to *Gapdh* of 2-3 experiments conducted in triplicate ± s.d., N = 6-9, Student's unpaired two-tailed t-test.

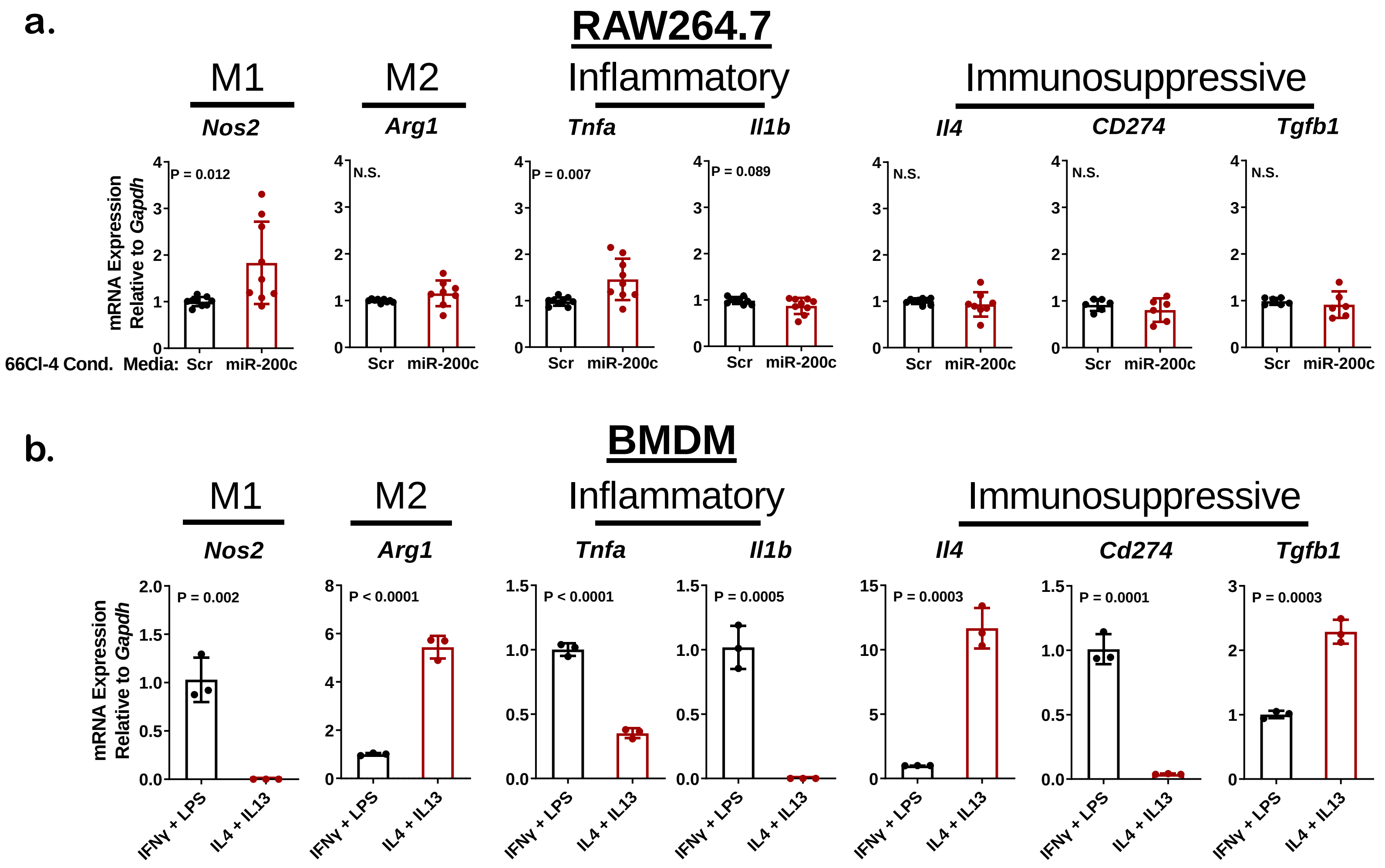

Supplementary Figure 6. a. 66Cl-4 cells were transiently transfected with Scramble control (Scr) or miR-200c mimic (miR-200c). After 96 hours, 66Cl-4 Scr or 66Cl-4 miR-200c conditioned medium (CM) was placed on RAW264.7 cells. At 48 hours culture with 66Cl-4 conditioned medium, macrophage polarization was determined via qRT-PCR for M1 (*Nos2* and pro-inflammatory cytokines: *Tnfa* and *Il1b*) and M2 (*Arg1* and immunosuppressive cytokines/markers: *Il4*, *Cd274*, *Tgfb1*) genes. Shown is the mean mRNA expression relative to *Gapdh* of 2-3 experiments conducted in triplicate ± s.d., N = 6-9, Student's unpaired two-tailed t-test. **b.** Bone marrow-derived macrophages (BMDM) were cultured in the presence of 25 ng/mL M-CSF for 5 days at which time M2 macrophage polarization was induced via 20 ng/mL IL4 and 20 ng/mL IL13. BMDM were similarly cultured with 5 ng/mL GM-CSF for 5 days at which time M1 macrophage polarization was induced via 20 ng/mL IFNγ and 100 ng/mL LPS. Macrophage polarization was confirmed on day 7 via qRT-PCR as in (**a**). Shown is the mean mRNA expression relative to *Gapdh* of an experiment conducted in triplicate ± s.d., N = 3, Student's unpaired two-tailed t-test.

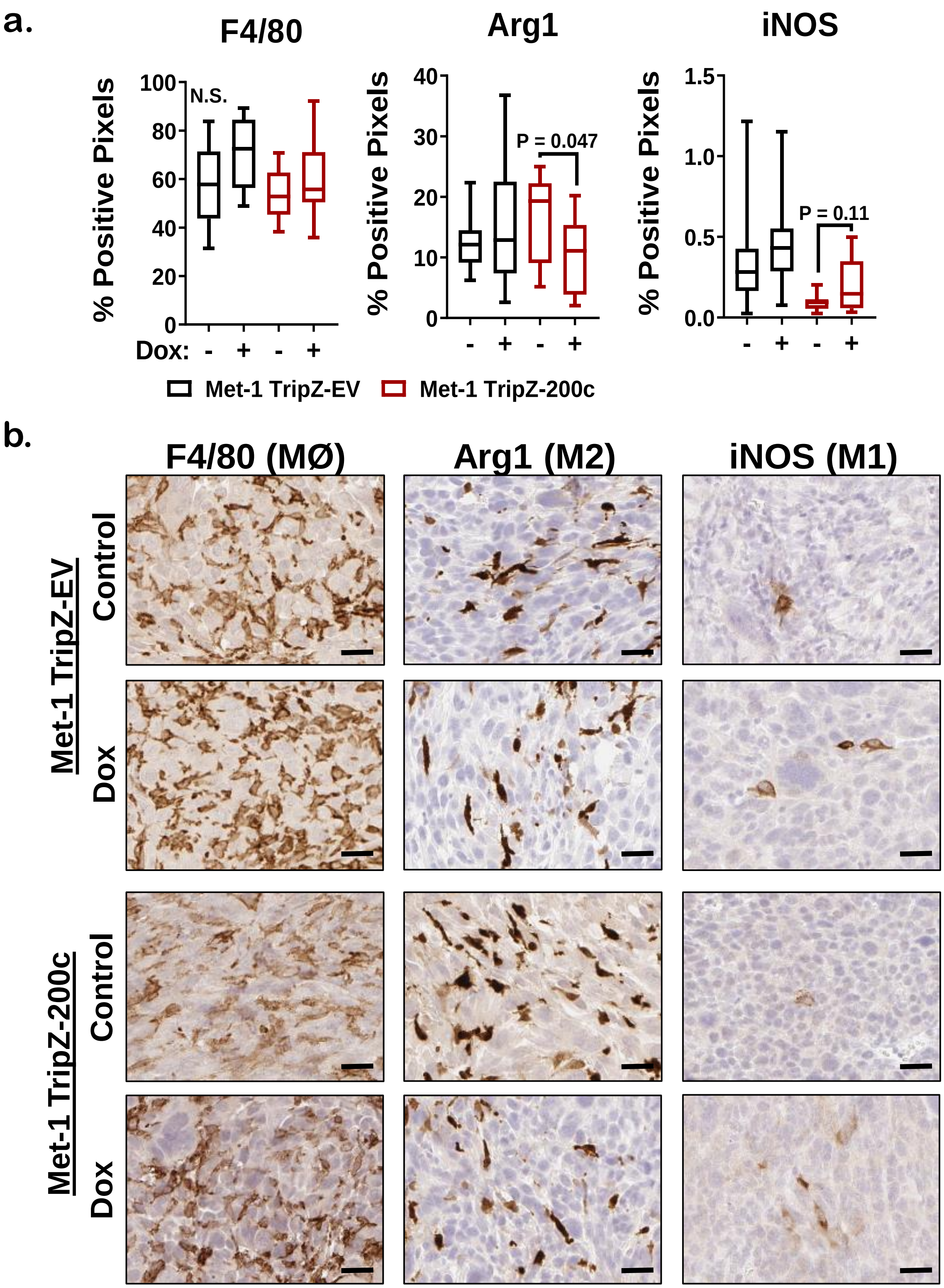

Supplementary Figure 7. a-b. IHC was conducted on Met-1 TripZ-EV and Met-1 TripZ-200c tumors. Quantification was done on the entirety of each tumor using the Aperio microscope and ImageScope software (**a**). Percent pixels are presented as a box plot with the center line representing the median, the box representing the 25th and 75th percentiles and the whiskers representing the minimum and maximum, N = 8-14, Student's unpaired two-tailed T-test. Representative images are shown (**b**), 10x, scale bar = 30 µm.

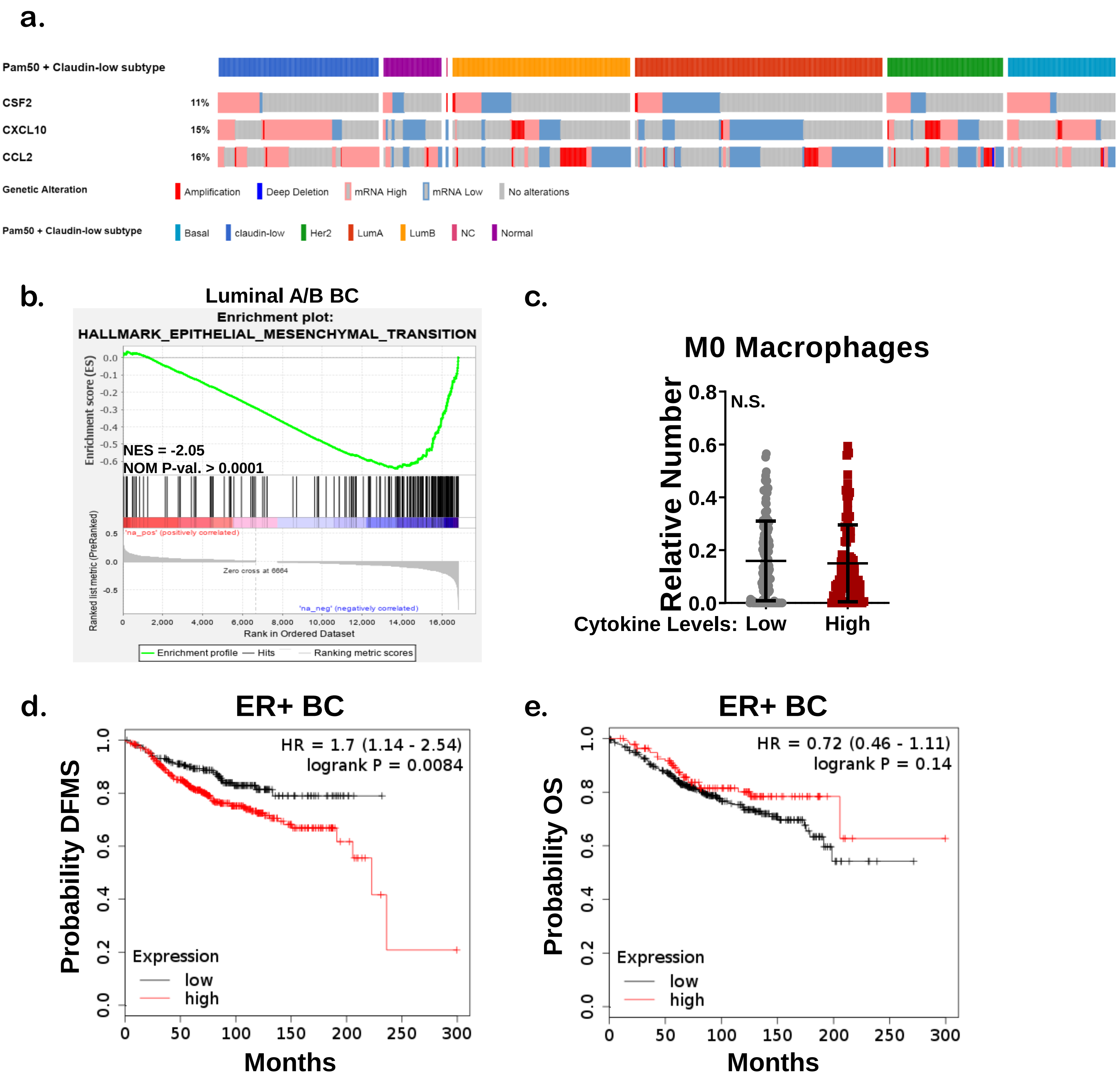

Supplementary Figure 8. a. Alterations in *CSF2* (GM-CSF), *CXCL10* and *CCL2* were determined using cbioportal (Nature 2012, METABRIC, Nature Communications 2016 datasets, z-score threshold ± 1.5, N = 1904). Percentages denote the total percent of patients with alterations in each gene. **b.** Genes that were enriched with *CSF2*, *CXCL10* and *CCL2* alterations according to the Luminal A/B dataset in the TCGA (Nature 2012) were subjected to GSEA analysis (Hallmark), NES = normalized enrichment score, NOM p-val = nominal p-value. **c.** The Firehouse Legacy dataset (TCGA) was assessed for the relative number of immune infiltrates using CIBERSORT. Patients with expression of each *CSF2*, *CXCL10* and *CCL2* in the lowest quartile (Low, N = 92) or highest quartile (High, N = 132) were stratified via the relative number of unactivated macrophages (M0). Shown is the mean ± s.d., Student's unpaired two-tailed t-test. **d-e.** KMplotter was used to stratify ER+ BC patients with high expression of *CSF2*, *CXCL10* and *CCL2* (red) based on disease free metastasis survival (DFMS, **d**, N = 664) and overall survival (OS, **e**, N = 2548).

Supplementary Figure 9. Uncropped western blot images from Figure 1b (**a**) and Supplementary Figure 5b (**b**).

a.

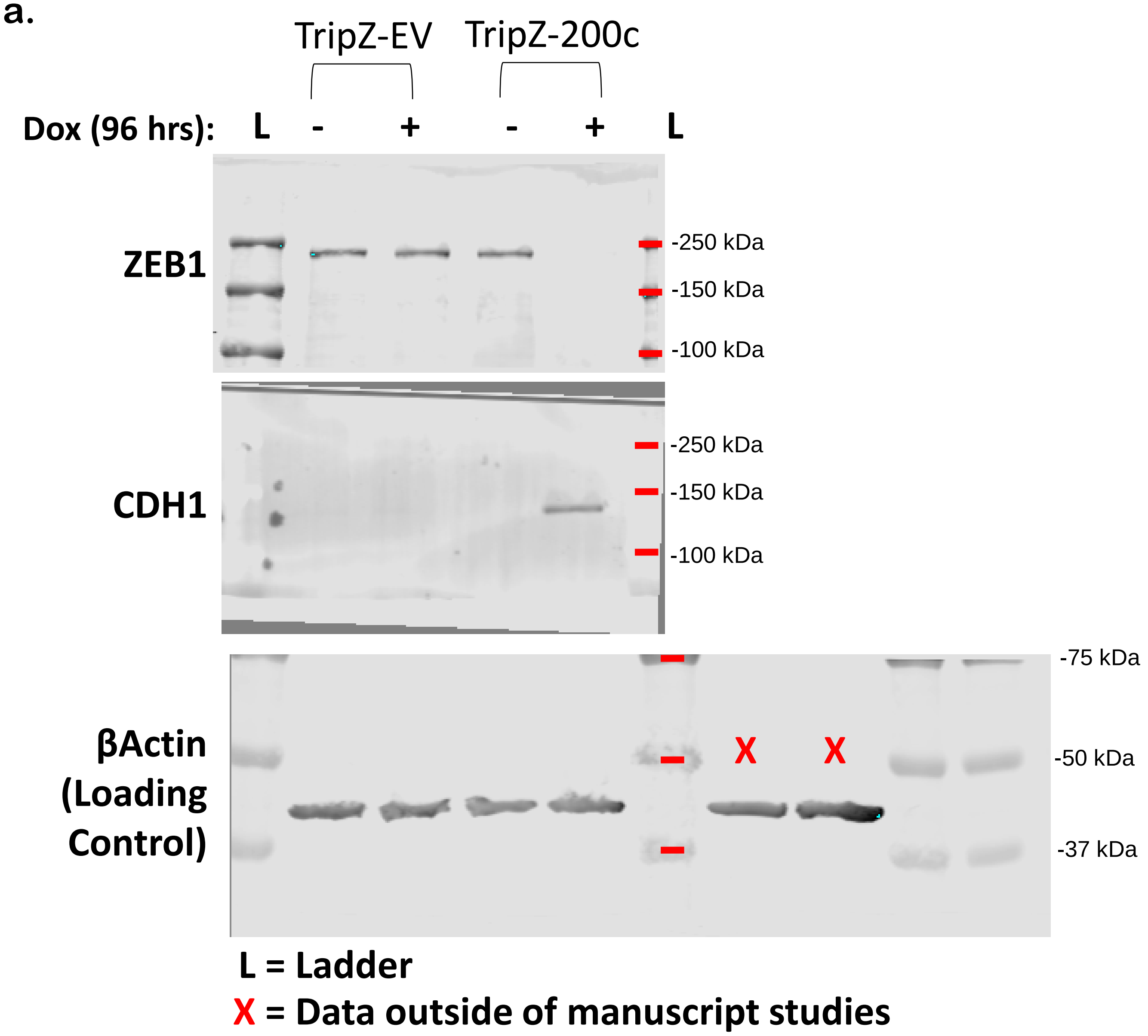

Supplementary Figure 9 (cont.). Uncropped western blot images from Figure 1b (**a**) and Supplementary Figure 5b (**b**).

b.

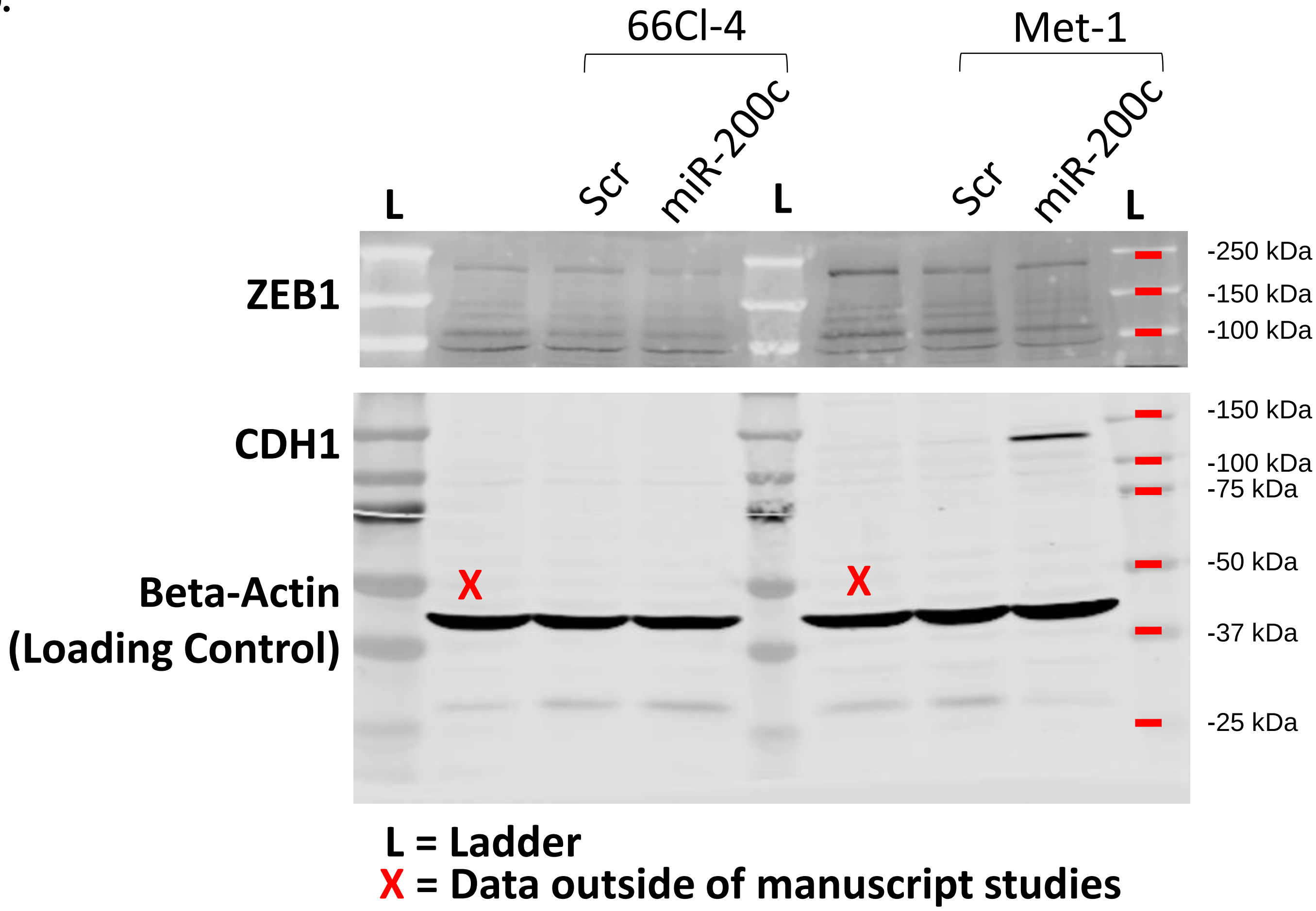

Supplement: Supplementary file 1 — Supplementary Information [file 41523_2021_273_MOESM1_ESM.pdf]
