# Supplementary material for: MicroRNA-200c restoration reveals a cytokine profile to enhance M1 macrophage polarization in breast cancer
Source: NPJ Breast Cancer. 2021 May 27;7:64. doi: 10.1038/s41523-021-00273-1 (PMC8160264; doi:10.1038/s41523-021-00273-1)
Supplement: Supplementary file 9 — Reporting Summary [file 41523_2021_273_MOESM9_ESM.pdf]

nature research

Corresponding author(s): Dr. Jennifer Richer

Last updated by author(s): Apr 22, 2021

Reporting Summary

Nature Research wishes to improve the reproducibility of the work that we publish. This form provides structure for consistency and transparency in reporting. For further information on Nature Research policies, see our Editorial Policies and the Editorial Policy Checklist.

Statistics

For all statistical analyses, confirm that the following items are present in the figure legend, table legend, main text, or Methods section.

n/a	Confirmed	
☐	☒	The exact sample size (*n*) for each experimental group/condition, given as a discrete number and unit of measurement
☐	☒	A statement on whether measurements were taken from distinct samples or whether the same sample was measured repeatedly
☐	☒	The statistical test(s) used AND whether they are one- or two-sided<br>*Only common tests should be described solely by name; describe more complex techniques in the Methods section.*
☐	☒	A description of all covariates tested
☐	☒	A description of any assumptions or corrections, such as tests of normality and adjustment for multiple comparisons
☐	☒	A full description of the statistical parameters including central tendency (e.g. means) or other basic estimates (e.g. regression coefficient) AND variation (e.g. standard deviation) or associated estimates of uncertainty (e.g. confidence intervals)
☒	☐	For null hypothesis testing, the test statistic (e.g. *F*, *t*, *r*) with confidence intervals, effect sizes, degrees of freedom and *P* value noted<br>*Give P values as exact values whenever suitable.*
☒	☐	For Bayesian analysis, information on the choice of priors and Markov chain Monte Carlo settings
☒	☐	For hierarchical and complex designs, identification of the appropriate level for tests and full reporting of outcomes
☒	☐	Estimates of effect sizes (e.g. Cohen's *d*, Pearson's *r*), indicating how they were calculated

Our web collection on statistics for biologists contains articles on many of the points above.

Software and code

Policy information about availability of computer code

Data collection	Microsoft Excel 2016, cellSens Standard Software (ver2.3), IncuCyte Zoom Software (ver2018A), ImageScope x64 Software (ver12.4.0), Applied Biosystems 7500 Software (ver2.3), Gen5 Software (ver2.0), cBioPortal, Image Studio Software (ver5.2)
Data analysis	ImageJ, GraphPad Prism (ver8), IncuCyte Zoom Software (ver2018A), ImageScope x64 Software (ver12.4.0) , Microsoft Excel 2016, GSEA (ver4.0.3), InForm Software (ver2.4), R, DAVID 6.8, CIBERSORT, Kaplan Meier-plotter, gSNAP, Cufflinks

For manuscripts utilizing custom algorithms or software that are central to the research but not yet described in published literature, software must be made available to editors and reviewers. We strongly encourage code deposition in a community repository (e.g. GitHub). See the Nature Research guidelines for submitting code & software for further information.

Data

Policy information about availability of data

All manuscripts must include a data availability statement. This statement should provide the following information, where applicable:

- Accession codes, unique identifiers, or web links for publicly available datasets
- A list of figures that have associated raw data
- A description of any restrictions on data availability

For any novel reagent or resource (e.g. Met-1 TripZ-EV or Met-1 TripZ-200c cell lines) created in the course of this project, we will follow the NIH policy on timely distribution and sharing on biomedical research resources, as published in the NIH Grants Policy Statement. As appropriate, sharing will be under the guidance of the CU Innovations technology transfer operations. mRNA sequencing data shown in Figure 1e-g, Supplementary Figure 1b-c and Supplemental Tables S1-6 are already deposited in NCBI's Gene Expression Omnibus (73) and are accessible through GEO Series accession number GSE151320 (https://www.ncbi.nlm.nih.gov/geo/query/acc.cgi?acc=GSE151320). Additional data files generated and analyzed during this study are described and shared in the following figshare data record: https://doi.org/10.6084/m9.figshare.14456310 (85).

Field-specific reporting

Please select the one below that is the best fit for your research. If you are not sure, read the appropriate sections before making your selection.

☒ Life sciences ☐ Behavioural & social sciences ☐ Ecological, evolutionary & environmental sciences

For a reference copy of the document with all sections, see nature.com/documents/nr-reporting-summary-flat.pdf

Life sciences study design

All studies must disclose on these points even when the disclosure is negative.

Sample size	When appropriate, tissue culture experiments are the mean of three separate experiments conducted in triplicate to decuple. For in vivo experiments, animal numbers were calculated at 80% power to the expected difference (based on published studies) at P < 0.05 (two-tailed).
Data exclusions	Mice that developed ulcers were excluded from the data analysis. For experiments with an N > 9, data points were excluded if they were two standard deviations above or below the mean.
Replication	All experiments, including animal experiments, were repeated at least once to ensure reproducibility.
Randomization	Microsoft Excel 2016 was used to randomly sort animals by number and mice were then divided equally into treatment groups.
Blinding	All investigators who performed data analysis and stastatics on animal experiments were blinded to study group.

Reporting for specific materials, systems and methods

We require information from authors about some types of materials, experimental systems and methods used in many studies. Here, indicate whether each material, system or method listed is relevant to your study. If you are not sure if a list item applies to your research, read the appropriate section before selecting a response.

Materials & experimental systems

n/a	Involved in the study	
☐	☒	Antibodies
☐	☒	Eukaryotic cell lines
☒	☐	Palaeontology and archaeology
☐	☒	Animals and other organisms
☒	☐	Human research participants
☒	☐	Clinical data
☒	☐	Dual use research of concern

Methods

n/a	Involved in the study	
☒	☐	ChIP-seq
☒	☐	Flow cytometry
☒	☐	MRI-based neuroimaging

Antibodies

Antibodies used	β-Actin, clone# 13E5, lot# 15, Cell Signaling Technology Cat# 4970, RRID:AB_2223172; CDH1, clone# 24E10, lot# 12, Cell Signaling Technology Cat# 3195, RRID:AB_2291471; ZEB1, lot# G115263, Sigma-Aldrich Cat# HPA027524, RRID:AB_1844977; Goat anti-Rabbit Secondary, lot# C90619-05, LI-COR Biosciences Cat# 925-32211, RRID:AB_2651127; anti-Rabbit Secondary, lot# 1705869 Thermofisher, A-11008; Ki67, Abcam Cat# ab15580, RRID:AB_443209; PyMT, Novus Cat# NB100-2749, RRID:AB_10001944; F4/80, clone# BM8, lot# 1711898, Thermo Fisher Scientific Cat# MF48000, RRID:AB_10376289; Arg1, clone# D4E3M, lot# 1, Cell Signaling Technology Cat# 93668, RRID:AB_2800207; iNOS, clone# D6B6S, lot# 5, Cell Signaling Technology Cat# 13120, RRID:AB_2687529; CD8, clone# 4SM15, lot# 4348256, Thermo Fisher Scientific Cat# 14-0808-80, RRID:AB_2572860
Validation	We confirmed that all antibodies detected the mouse protein of interest by conducting a titer via immunohistochemistry on positive mouse tissues, as recommended by each manufacturer.

Eukaryotic cell lines

Policy information about cell lines

Cell line source(s)	Mouse mammary carcinoma cells were kindly provided by Drs. Donald McDonnell and Alexander Borowsky. RAW264.7 cells were kindly provided by Dr. Phillip Owens. Human breast cancer cells were purchased from the ATCC (BT549 in 2008) or from the University of Colorado Cancer Anschutz Medical Campus Cell Culture Services Core (SUM159PT in 2013).
Authentication	Human cell lines were short Tandem Repeat Fingerprinted by the University of Colorado Anschutz Medical Campus Cell Culture Services Core and had 100% match to ATCC for BT549 cells and Asterand for SUM159PT cells (August 2017).

Mycoplasma contamination	Cells were tested for mycoplasma contamination every three months and prior to use in in vivo experiments. The MycoAlert PLUS mycoplasma detection kit (Lonza, Cat# 75860-358) was used.
Commonly misidentified lines (See ICLAC register)	N/A

Animals and other organisms

Policy information about studies involving animals; ARRIVE guidelines recommended for reporting animal research

Laboratory animals	6-8 week old female FVB/NJ mice (IMSR Cat# JAX:001800, RRID:IMSR_JAX:001800)
Wild animals	N/A
Field-collected samples	N/A
Ethics oversight	All animal experiments were performed in accordance with international, national and institutional guidelines for humane research under a protocol (#00407) approved by the University of Colorado Institutional Animal Care and Use Committee (IACUC).

Note that full information on the approval of the study protocol must also be provided in the manuscript.
